# Cholangiocyte organoids from human bile retain a local phenotype and can repopulate bile ducts in vitro

**DOI:** 10.1002/ctm2.566

**Published:** 2021-12-26

**Authors:** Floris J. M. Roos, Haoyu Wu, Jorke Willemse, Ruby Lieshout, Laura A. Muñoz Albarinos, Yik‐Yang Kan, Jan‐Werner Poley, Marco J. Bruno, Jeroen de Jonge, Richard Bártfai, Hendrik Marks, Jan N. M. IJzermans, Monique M. A. Verstegen, Luc J. W. van der Laan

**Affiliations:** ^1^ Erasmus MC Department of Surgery, University Medical Center Rotterdam Rotterdam The Netherlands; ^2^ Department of Molecular Biology, Radboud University Nijmegen The Netherlands; ^3^ Erasmus MC Department of Gastroenterology and Hepatology, University Medical Center Rotterdam Rotterdam The Netherlands

**Keywords:** bile, cholangiocyte‐organoids, cholangiocytes, ERCP, extrahepatic bile duct, gallbladder, regeneration, repopulation

## Abstract

The well‐established 3D organoid culture method enabled efficient expansion of cholangiocyte‐like cells from intrahepatic (IHBD) and extrahepatic bile duct (EHBD) tissue biopsies. The extensive expansion capacity of these organoids enables various applications, from cholangiocyte disease modelling to bile duct tissue engineering. Recent research demonstrated the feasibility of culturing cholangiocyte organoids from bile, which was minimal‐invasive collected via endoscopic retrograde pancreaticography (ERCP). However, a detailed analysis of these bile cholangiocyte organoids (BCOs) and the cellular region of origin was not yet demonstrated. In this study, we characterize BCOs and mirror them to the already established organoids initiated from IHBD‐ and EHBD‐tissue. We demonstrate successful organoid‐initiation from extrahepatic bile collected from gallbladder after resection and by ERCP or percutaneous transhepatic cholangiopathy from a variety of patients. BCOs initiated from these three sources of bile all show features similar to in vivo cholangiocytes. The regional‐specific characteristics of the BCOs are reflected by the exclusive expression of regional common bile duct genes (*HOXB2* and *HOXB3*) by ERCP‐derived BCOs and gallbladder‐derived BCOs expressing gallbladder‐specific genes. Moreover, BCOs have limited hepatocyte‐fate differentiation potential compared to intrahepatic cholangiocyte organoids. These results indicate that organoid‐initiating cells in bile are likely of local (extrahepatic) origin and are not of intrahepatic origin. Regarding the functionality of organoid initiating cells in bile, we demonstrate that BCOs efficiently repopulate decellularized EHBD scaffolds and restore the monolayer of cholangiocyte‐like cells in vitro. Bile samples obtained through minimally invasive procedures provide a safe and effective alternative source of cholangiocyte organoids. The shedding of (organoid‐initiating) cholangiocytes in bile provides a convenient source of organoids for regenerative medicine.

## Graphical abstract

**Lay summary**: Cholangiocyte organoids provide a powerful tool for characterizing bile duct epithelium and expanding cholangiocytes for tissue engineering purposes. However, organoid initiation typically requires invasive tissue‐biopsies obtained via surgical procedures associated with complications. To overcome this limitation, organoid cultures were initiated from minimal invasive bile‐samples obtained during routine clinical procedures. Characterization revealed that these bile‐cholangiocyte organoids (BCOs) originate from extrahepatic biliary tissue and are capable of repopulating human extrahepatic bile duct scaffolds. Upon repopulation of scaffolds in vitro, the cells obtain a transcriptomic profile more closely resembling primary cholangiocytes. By using bile as novel source for extrahepatic cholangiocyte organoids, bile duct tissue engineering as well as personalized disease modelling become feasible.

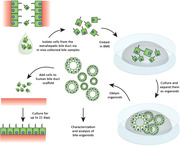

## 1 INTRODUCTION

The biliary tree is a complex tubular system representing the extrahepatic bile duct (EHBD) and the intrahepatic bile duct (IHBD).^1^ In addition to the difference in anatomical location, both these ductal structures originate from distinct progenitor cells during embryogenic development.^2^ The IHBD arises from bipotential hepatoblasts, while the EHBD develops from a shared pancreaticobiliary progenitor.^2^, ^3^ Despite the divergent origin, a single layer of highly specialized epithelial cells, cholangiocytes, are lining the ducts of the entire biliary tree.^1^ Cholangiocytes are responsible for modifying bile composition and form an important barrier between the cytotoxic bile and surrounding tissues. Defects in cholangiocytes can result in severe diseases (cholangiopathies), often developing into end‐stage liver diseases, for which liver transplantation is the only curative therapy.^3^ The regional diversity present in cholangiocytes is also reflected in different cholangiopathies.^4^, ^5^ For instance, Alagille syndrome is only affecting intrahepatic cholangiocytes, in line with the underlying autosomal mutation that prevents proper hepatoblast differentiation towards IHBDs. Other examples are primary sclerosing cholangitis (PSC) and non‐anastomotic bile duct strictures, diseases that are predominantly affecting the EHBD.^4^, ^5^, ^6^ The presence of regional diversity in the extrahepatic biliary tree was recently confirmed by Rimland et al., where the authors showed distinct gene‐expression profiles in cholangiocytes covering different parts of the EHBD.^7^ In this study, 3D cholangiocyte organoids were cultured from both IHBD and EHBD and it is demonstrated that only intrahepatic cholangiocyte organoids (ICOs) could (partially) differentiate towards hepatocyte‐like cells. This result reflects the embryogenic origin of this type of cholangiocytes.^7^ Initially, cholangiocyte organoids were initiated from liver biopsies, and described as liver‐derived bipotent stem cells in vitro.^8^ However, elaborated studies demonstrate that mature cholangiocytes, undergoing widespread (epi)genetic remodelling into a highly proliferative state, are the organoid‐initiating cell type but not adult (biliary) stem cells. Due to canonical Wingless‐related integration site (WNT)‐stimulating culture conditions, cholangiocyte organoids start expressing adult stem cell markers/WNT‐target genes (*LGR5*), which are substantially overexpressed when compared to in vivo cholangiocytes.^9^, ^10^, ^11^, ^12^ By doing so, cholangiocyte organoids acquire a phenotype comparable to rapid proliferating cholangiocytes in vivo (ductal reprogramming).

As ICOs maintain patient‐specific characteristics upon expansion, they provide a powerful tool to study the biology and pathophysiology of cholangiocytes.^8^ The downside of this culture platform is that ICOs are generally initiated from liver biopsies which are collected during potentially harmful interventions, or during liver transplantation. This limits the use of COs in disease modelling and studying disease progression to a limited subset of patients, that is, patients who undergo liver transplantation due to end‐stage liver disease. To overcome these hurdles, an elegant and minimally invasive alternative to initiate patient‐specific organoids was shown by Soroka et al.^13^ Here, the authors expanded bile cholangiocyte organoids (BCOs) from bile samples collected during routine clinical procedures via endoscopic retrograde cholangiopancreaticography (ERCP).^13^ However, it is still unclear from which region in the biliary tree (intra‐ or extra‐hepatic) the BCO‐initiating cells originate from. Furthermore, a recent study demonstrated that cholangiocyte organoids derived from human EHBD and cultured in non‐canonical WNT‐stimulating conditions could efficiently repopulate collagen scaffolds that were successfully transplanted into mice as functional EHBDs.^14^ However, whether this is feasible with patient‐derived COs cultured in canonical WNT‐stimulating conditions obtained from in vivo collected bile is still unknown.

Therefore, the aim of our study is to further characterize the properties of BCOs with a focus on determining the anatomical origin of BCOs. Moreover, in addition to ERCP as a source of BCOs, we extent the sources of bile with collection via percutaneous transhepatic cholangiopathy drainage (PTCD) and directly from resected gallbladders. As a proof of principle, we show efficient repopulation of human bile duct scaffolds using BCOs collected via ERCP from patients and demonstrate that human EHBD scaffold can help sustain organoids to form a functional cholangiocyte monolayer in vitro. Based upon this evidence, the feasibility for the use of BCOs in personalized regenerative medicine is near.

## 2 MATERIALS AND METHODS

### 2.1 Bile, brush and tissue collection

Bile samples (1 ml per patient) were obtained in vivo from patients suffering from biliary diseases (Table 1 and Table S1) and which were undergoing ERCP (*n* = 54) or PTCD (*n* = 3) for regular treatment regimens at the Erasmus MC, Rotterdam, the Netherlands. An additional brush specimen (*n* = 2) was collected from the common bile duct (CBD) via ERCP if the patient already underwent brush cytology for standard diagnostics. Bile samples collected in vivo were transported at 4˚C and processed within 1 h after collection. All patients gave written informed consent to use the bile collected during these procedures for research purposes. This study was approved by the local Erasmus MC Medical Ethical Committee and registered under number MEC‐2016‐743.

**TABLE 1:** Patient and organoid culture characteristics

Culture type	Age patient (years)	Gender	Cell source	Donor type or indication ERCP/surgery	Freezed in after passage
ECO^a^ 8	13	M	GB	DBD	5
BCO 1, ICO 1, ECO 1	27	M	Liver, EHBD, GB bile	DBD	8
BCO 5	33	F	ERCP bile	Bile leakage	18
BCO 6	46	M	ERCP bile	PSC	26
ECO^a^ 9	50	M	GB	Bile stones	5
BCO 3, ICO 3, ECO 3	56	F	Liver, EHBD, GB bile	Liver explant of a cirrhotic PSC liver	10
ECO^a^ 10	59	M	GB	DBD	5
BCO 2, ICO 2, ECO 2	59	F	Liver, EHBD, GB bile	DBD	10
BCO 4, ICO 4, ECO 4	66	F	Liver, EHBD, GB bile	Liver explant of cirrhotic HCV liver with HCC	6
BCO 7	68	F	ERCP bile	Bile stones	18

**Abbreviations**: AS, anastomotic bile duct stricture; BCO, bile cholangiocyte organoid; DBD, donation after brainstem death; ECO, extrahepatic cholangiocyte organoids; EHBD, extrahepatic bile duct; ERCP, endoscopic retrograde cholangiopancreaticography; F, female; GB, gallbladder; GB bile, gallbladder bile; HCC, hepatocellular carcinoma; HCV, hepatitis C virus; ICO, intrahepatic cholangiocyte organoids; M, male; PSC, primary sclerosing cholangitis.

^a^ Indicates that these organoids are cultured using the previously published protocol^14^ stimulating non‐canonical WNT culture conditions.

Bile (3 ml) was collected from gallbladders from donor (*n* = 6) or explanted livers (*n* = 8) obtained during liver transplantation procedures performed at the Erasmus MC, Rotterdam and was stored at 4˚C and processed within 24 h after collection. All patients or next of kin gave written informed consent to use the tissue for research purposes. This study was approved by the local MEC of the Erasmus MC under number MEC‐2014‐060.

Tissue biopsies from liver (circa 0.5–3 cm^3^, *n* = 4), extrahepatic bile duct (EHBD, circa 0.5–3 cm^3^, *n* = 4) and gallbladders (*n* = 3, scraped cells) were obtained from donor livers (*n* = 5) or explant livers (*n* = 2) during liver transplant procedures performed at the Erasmus MC, Rotterdam, the Netherlands. For complete methodology, see Supplementary Materials and Methods.

### 2.2 Initiation and culture expansion of organoids

Organoids from liver‐ and EHBD biopsies were processed, initiated and expanded as previously published by Rimland et al.^7^ and Huch et al.^8^ For detailed methodology and culture conditions, see Supplementary Materials and Methods and Table S2.

Organoids from bile (BCOs) were initiated according to an adapted protocol from Soroka et al.^13^ for culturing BCOs from bile collected via ERCP. For detailed methodology and culture conditions, see Supplementary Materials and Methods and Table S2. All experiments were performed with passage five organoids, unless otherwise stated. When referred to ECO‐cultures in experiments, these cultures resemble canonical WNT‐stimulated ECOs (tissue obtained according to Rimland et al. and cultured in the Huch et al. conditions, Table S2),^7^, ^8^ unless otherwise stated.

### 2.3 Flow cytometry

Flow cytometry analysis was performed to evaluate the presence and frequency of EPCAM^+^ cells in the collected bile samples from ERCP patients. Bile cells were stained with antibodies against human CD326 (EpCAM) (Biolegend 324203; mouse monoclonal – FITC conjugated, 1:100) according to the manufacturer's instructions. Flow cytometry analysis was performed using a Canto flow cytometer (BD Biosciences) and cell populations were analysed using Flowjo (version v10.6.1, BD).

### 2.4 RNA extraction, cDNA synthesis and RT‐qPCR

Total RNA was collected after removal of the culture medium by adding 700 μl of QIAzol lysis reagent (Qiagen) to a 24 well containing organoids (two 25 μl basement membrane extract ‐BME‐/matrigel domes). RNA was isolated using the miRNeasy kit (Qiagen) according to the manufacturer's protocol^15^ and the concentration was measured using a NANOdrop 2000 (ThermoFisher). cDNA from 500 ng RNA was prepared using 5x PrimeScript RT Master Mix in a 2720 thermal cycler (Applied Biosystems). RT‐qPCR was performed with the primer sets provided in Table S3. RT‐qPCR data are presented as mean with a 95% confidence interval or as standard error of the mean. RT‐qPCR values are relative to the housekeeping gene Glyceraldehyde‐3‐Phosphate Dehydrogenase *(GAPDH)* or Hypoxanthine‐guanine‐fosforibosyl‐transferase *(HPRT)*.

### 2.5 Immunofluorescence staining

To evaluate protein expression of the organoids, immunofluorescence (IF) was performed with selected antibodies for cytokeratin (KRT)‐7, KRT‐19, SRY box (SOX)9, Albumin, mucin‐1 (MUC‐1), secretin receptor (SCTR) and cystic fibrosis transmembrane conductance regulator (CFTR) (complete list of antibodies and dilutions used is displayed in Table S4), as previously described for ICOs.^8^ For detailed methodology, see Supplementary Materials and Methods.

### 2.6 Ussing chamber assay

COs cultured from tissue and bile were seeded on a transwell culture plate (24 well plate 6.5 mm, Corning) to grow COs in a 2D fashion. Upon forming a confluent monolayer, transwells were placed in an Ussing chamber (Physiologic Instruments, San Diego, CA, USA) set up to analyse functional cholangiocyte‐specific transporter channels (CFTR and Ca^2+^‐activated Cl^–^ channel) using Acquire & Analyze Software 2.3 (Physiologic Instruments). For detailed methodology, see Supplementary Materials and Methods.

### 2.7 Glutamyltransferase assay

Supernatant (10 μl) of BCOs (*n* = 3) and, as a positive control, of ECOs cultured in non‐canonical WNT conditions (*n* = 3), was collected. These specific ECOs were chosen since they were shown to excrete the same amount of ϒ‐glutamyltransferase (GGT) as primary cholangiocytes.^14^ GGT activity was determined using a colorimetric assay kit (MAK089; Sigma‐Aldrich), performed according to the manufacturer's protocol.

### 2.8 Rhodamine‐123 transport functionality

Functionality of the multidrug resistance (MDR)‐1 transporter was assessed using the Rhodamine‐123 assay.^16^ Specificity was determined by blocking MDR‐1 transporter with Verapamil (10 μM, Sigma Aldrich) for 30 min at 37˚C prior to Rhodamine‐123 incubation (100 μM, Sigma Aldrich). Subsequent confocal images were acquired using a Leica SP5 confocal microscope (LEICA) equipped with a 488 nm laser and a 20x zoom dipping lens.

### 2.9 Cell proliferation assessment

Cell proliferation characteristics of BCOs, ICOs and ECOs were measured by PrestoBlue Metabolic Assay (Life Technologies) and 5‐Ethynyl‐2′‐deoxyuridine (EdU)‐incorporation (ThermoFisher). For detailed methodology and gating strategy, see Supplementary Materials and Methods and Figure S3A and B.

### 2.10 RNA sequencing data obtaining and analysis

RNA was isolated from three biological replicates from different types of organoids (ERCP bile, GB bile, ICO and ECO). Five hundred nanograms of total RNA was used for library construction using KAPA RNA Hyper + RiboErase HMR kit (Roche 08098140702). RNA‐sequencing (RNAseq) libraries were sequenced paired‐end (2×38bp) on Illumina NextSeq 500 platform. After sequencing, the qualities of the reads were checked using fastqc. An average of 20 million paired‐end reads were aligned to human genome (hg38) using STAR^17^ (2.7.0f) with default settings. BAM files were sorted and indexed using SAMtools^18^ (1.7). The number of mapped fragments was quantified at gene level using featurecount^19^ (1.6.4) with the following parameters, ‐p ‐g gene_name, based on Gencode annotation (v30). The R package DESeq2^20^ (1.22.2 on R 3.5.1) was used for differential gene expression analysis and generating principal component analysis (PCA) and heatmap plots. Genes with less than two fragments were removed from the differential gene expression analysis. All the differentially expressed genes are determined according to adj‐*p*<0.05. Vst normalized counts were generated using vst function from DESeq2, and transformed to Z‐scores. Pheatmap package was used to generate heatmap plots using either vst normalized counts or Z‐scores as input. Pathway analyses were performed by Enrichr web tool^21^ using the significantly differentially expressed genes as input with default settings. RNAseq data from gallbladder‐ and CBD tissue as published by Rimland et al.^7^ was used as a reference gene set for cholangiocytes and RNAseq data from 2D cultured primary hepatocytes was obtained from Schneeberger et al.^22^ as a reference gene set for hepatocyte gene‐expression. For comparing the transcriptome between the ERCP‐BCOs cultured in 3D (hydrogel) and ERCP‐BCOs that were cultured on scaffolds, RNA samples were isolated from three biological replicates. The library construction was done as mentioned before, and the libraries were sequenced pair‐end (2×42bp). The analysis was performed using the same strategy as mentioned before, but using the tools with different versions (STAR version = 2.7.6a, SAMtools version = 1.7, featureCounts version = 2.0.1, R version = 3.6.1 and DEseq2 version = 1.26.0). In particular, before generating the count table, subsampling was applied to have the samples with comparable sequencing depths using SAMtools. Differentially expressed genes are determined based on adj‐*p*<0.05 and |log2(Fold change) | > 1. The R package fgsea^23^ (1.12.0) was used for gene set enrichment analysis (GSEA). Genes upregulated in either 3D (hydrogel, BME) or scaffold culture system were used as input, and then compared to the differentially gene set published by Rimland et al.^7^ (tissue vs. organoid of CBD). Cell cycle‐related genes (involved in G2M and S phases) from R package Seurat (3.2.3)^24^ were used for the heatmap. RNAseq data from this study was disposed to the GEO dataset (number: GSE156519).

### 2.11 Upregulation of hepatocyte‐related genes

BCOs, ICOs and ECOs (from the same patient, *n* = 3 individual patients) were differentiated towards hepatocyte‐faith. The hepatocyte differentiation protocol used was adapted from Huch et al.^8^ with slight modifications (Clevers et al. patent: WO2017149025A1).^25^ For detailed methodology, see Supplementary Materials and Methods.

### 2.12 Repopulation of EHBD scaffolds

Decellularized human EHBD scaffolds were reseeded with BCOs. For this, EHBD tissue (*n* = 3, length: 4 cm) was obtained from research livers that were deemed unsuitable for transplantation. EHBD scaffolds were created according to the previously published protocol by Willemse et al.^26^ using a Triton‐X‐100‐based decellularization method. EHBD scaffolds were reseeded with single‐cells derived from ERCP‐BCOs (passage 5–9) and cultured for 21 days. For detailed methodology, see Supplementary Materials and Methods.

### 2.13 Statistical analyses

All statistical analyses were conducted using SPSS (software version 21, SSPS Inc, Chicago, IL, USA). Qualitative data were analysed with the χ2 or Fisher exacts tests and were presented with numbers and percentages. Continuous variables were tested using an independent *T*‐test or Mann–Whitney‐U test and presented with normal distribution as means with standard deviation and if not normally distributed, they are presented as range. In all tests, a *p* value of <0.05 is considered significant.

## 3 RESULTS

### 3.1 Human bile harbours organoid‐initiating cells

**FIGURE 1:**
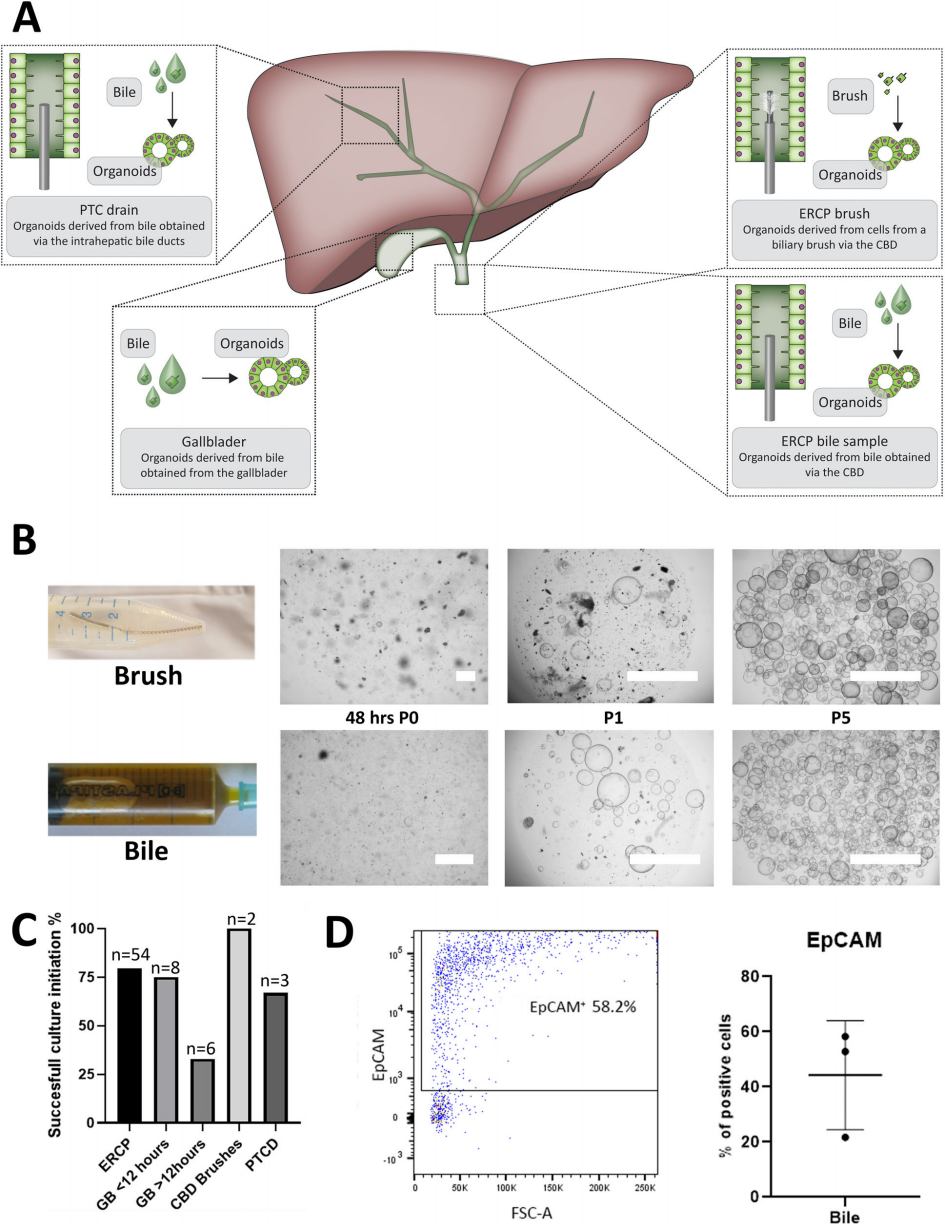
Culture of bile cholangiocyte organoids. (A) Schematic overview of initiation of bile cholangiocyte organoids (BCOs) from different sources of bile and from endoscopic retrograde cholangiopancreatography (ERCP)‐brushes of the common bile duct (CBD). (B) Representative organoid cultures from ERCP‐brushes and ERCP‐derived bile at different passages. Passage 0 (48 h after culture initiation), P1 (picture taken 72 h after passage 1) and P5 (picture taken 120 h after passage 5) (p), scale bars indicate 1000 μm. (C) Successful culture percentage from different sources of bile and CBD brushes. (D) Flow cytometry analysis of cells within ERCP‐derived bile (*n* = 3) revealed that a mean 44.3%±19.7 is of biliary (EpCAM^+^) origin

Human bile was collected using the ERCP or PTCD procedure or from gallbladders after resection (Figure 1A, B). After obtaining and washing the cell fraction from bile, organoid cultures were initiated. Furthermore, cells obtained by CBD brushes were included and cultured as organoids.

BCOs could be initiated from healthy individuals as well as from a variety of patients with different underlying biliary diseases (Table 1 and Table S1), although with mixed success rates between sources. Upon initial issues with bacterial infections, vancomycin was added to the culture medium to prevent the loss of cultures without losing viable cells (Figure S1). In addition, to prevent fungal infections, which were frequently observed in patients suffering from PSC or cholangiocarcinoma (CCA), BCO cultures of these patients were supplemented with 1% antibiotic‐antimycotic instead of vancomycin for the first 3 days, as previously published.^13^ As shown in Figure 1C, after addition of treatment for micro‐organisms, organoid‐initiation was successful in 78% (42/54) of bile samples from ERCP. The success rate for brushes was 100% (2/2) and for bile samples from PTCD 67% (2/3). Organoids collected from ex vivo bile (resected gallbladders, >12 h after surgery) could be cultured with a lower 33% (2/6) success rate. This is possibly due to the longer storage time of the bile as compared to the rapidly processed ERCP and PTCD bile (cultured within 1 h after collection). Indeed, gallbladder bile processed within 12 h after resection had a 75% (6/8) success rate of BCO initiation (Figure 1C) and ERCP‐derived bile stored for 4 h before processing yielded no viable organoids (Figure S2). Furthermore, we could not find any difference in successful culture percentages between different underlying (biliary) conditions or between cultures derived from livers transplanted after brain death or circulatory death donation. As shown in Figure 1B, organoids from bile and brushes have a cystic morphology, similar to previous publications of organoids cultured from IHBD or EHBD tissue.^7^, ^8^ BCOs could be passaged long‐term (>passage 15, >5 months) and could be viably frozen at different time points (Table 1). Previous research showed for ICOs that the organoid‐initiating cells are EpCAM^+^ cells and likely a subset of cholangiocytes.^8^ As shown in Figure 1D, approximately 50% (mean 44.27% ±19.74, *n* = 3) of the cells present within bile are EpCAM^+^. These EpCAM^+^ cells in bile are the likely organoid‐initiating cells; however, direct evidence is still lacking. Direct cells sorting of EpCAM^+^ or EpCAM^–^ cells from bile using flow cytometry failed to yield viable cells for organoid‐initiation, thus indicating that likely due to cell stress‐related difficulties, we are unable to grow organoids from EpCAM^+^ cells from bile (Figure S2C). In summary, human bile samples are an effective source for cholangiocyte organoids.

### 3.2 Bile cholangiocyte organoids resemble functional cholangiocyte‐like cells in vitro

**FIGURE 2:**
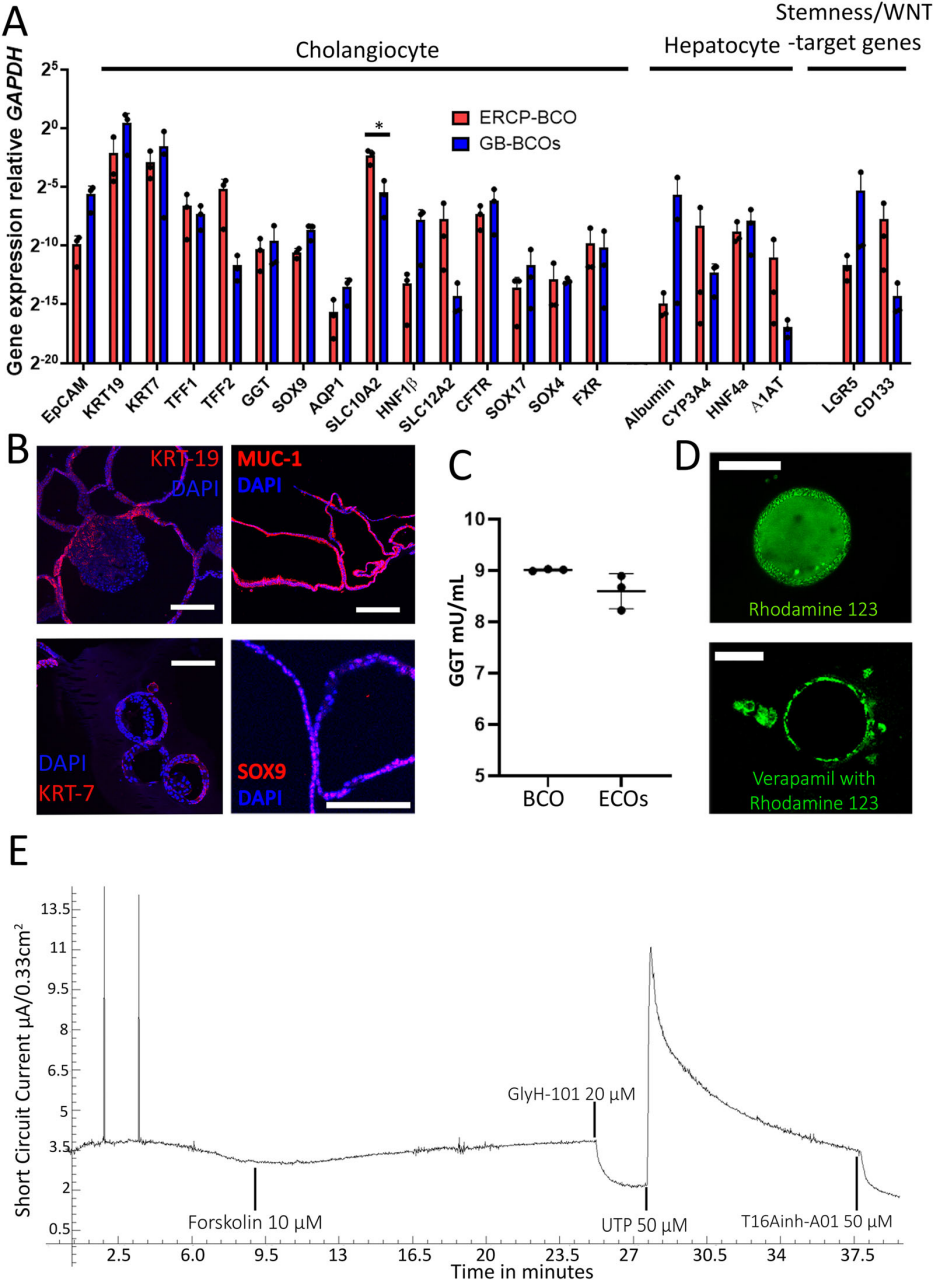
Bile cholangiocyte organoids represent functional cholangiocyte‐like cells in vitro. (A) Expression of typical cholangiocyte, hepatocyte and stemness/WNT‐target associated genes relative to *GAPDH* (presented as 2^–dct^ method) analysed using RT‐qPCR in BCOs (*n* = 6, BCO1‐3 and BCO5‐7). Mature cholangiocyte markers (*KRT7, KRT19*, *TFF1* and *TFF2*) were expressed relatively high compared to the expression of hepatocyte and stemness/WNT‐target genes. (B) Protein expression by immunofluorescence of the cholangiocyte markers: KRT‐19, KRT‐7, MUC‐1 and SOX9 (red) and nuclei (DAPI, blue) on BCOs (*n* = 3, BCO1, 5 and 7), scale bars indicate 100 μm except for SOX9, where it indicates 200 μm. Images displayed are from BCO 5 and 7. (C) Gamma‐glutamyltransferase activity of BCO supernatant as measured by fluorescence is similar between BCOs (*n* = 3, BCO5‐7) and ECOs (ECO8‐10, *n* = 3) grown in non‐canonical WNT‐stimulating conditions.^14^ (D) BCOs (*n* = 3, BCO1, 5 and 7) have clear MDR‐1 activity as Rhodamine 123 was actively transported out of the cells into the lumen of the organoid. Specificity was confirmed by inhibition with Verapamil, scale bars indicate 100 μm. (E) Representative ion‐channel functionality of 2D‐grown BCOs (*n* = 4, BCO1‐3 and 5) in an Ussing chamber. Stimulation with cAMP‐activator forskolin (both sides) resulted in an increase in short circuit current. This was completely blocked by cystic fibrosis transmembrane conductance regulator (CFTR)‐inhibitor, GlyH‐101, demonstrating specific CFTR‐mediated activity. It also shows calcium‐dependent chloride excretion ion‐channel activity, specifically stimulated by UTP and inhibited by T16Ainh‐A01, indicating anoctamin‐1 activity. *Indicates a significant difference (*p*<0.05)

All bile‐cholangiocyte cultures from either ERCP (*n* = 3) or gallbladder (*n* = 3) were analysed on gene‐expression by RT‐qPCR and normalized read counts from RNA‐seq data of BCOs compared to open access data from primary cholangiocytes and hepatocytes.^7^, ^22^ Overall, it is clear that the BCOs follow the transcriptomic profile of cholangiocytes and have limited expression of classical hepatocyte markers compared to primary hepatocytes (Figure S4A–C). Typical biliary markers, such as *KRT7*, *KRT19*, sodium‐depended bile acid transporter (*ASBT*, also known as *SLC10A2*), *SOX9*, *CFTR*, hepatocyte nuclear factor (*HNF*)‐*1β* and Trefoil factor (*TFF*)*1* and *TFF2*, were detected (Figure 2A and S4A). Interestingly, ERCP‐derived BCOs (bile collected from the CBD) expressed *TFF2* at somewhat higher levels when analysed by RNA‐seq, while gallbladder‐derived BCOs did not, but showed more expression of *SOX17* (Figure 2A and S4A and B). These results are in line with a recent publication demonstrating the local differences between cholangiocytes.^27^ Gallbladder‐BCOs had a high expression of *Albumin* (2/3 samples) and a low expression of *CYP3A4*, while ERCP‐derived BCOs show the opposite expression profiles (Figure 2A and S4C). Although this high expression of *Albumin* in GB‐BCOs could not be confirmed by RNA‐seq analysis and did not reach statistical significance, a difference between ERCP‐ and GB‐obtained BCOs still seems to be present (Figure S4C). In addition, ERCP‐BCOs showed higher expression of the apical *SLC10A2‐*transporter and the luminal *SLC12A2––*also known as *NKCC1*‐receptor when compared to GB‐BCOs (Figure 2A and S4A). Additionally, both BCOs express the WNT‐target gene leucine‐rich‐repeat‐containing‐G‐protein‐coupled receptor (*LGR*)*5* (Figure 2A and S4A) known to be higher expressed in cholangiocyte organoids compared to primary cholangiocytes (Figure S4C).^9^, ^11^ Overall, it is clear that BCOs not fully represent mature cholangiocytes, which is reflected in less expression of functional markers *AQP1*, *GGT* and *CFTR* (Figure S4A), similar to previous results.^7^ IF revealed protein expression of KRT‐7, KRT‐19, CFTR, MUC‐1, SCTR and SOX9 (Figure 2B and S4D), but the absence of ALB (Figure S4E) in BCOs. In addition, the functionality of BCOs was assessed by determining GGT activity, multidrug resistance protein‐1 (MDR1) activity and ion channel activity. Figure 2C demonstrates that GGT expression levels of BCOs were similar to those found in ECOs cultured in non‐canonical WNT‐stimulating conditions (mean 9.01±SD0.2 vs. mean 8.60±SD0.13, *p* = 0.1). Previous published data showed that these specific ECOs have a similar GGT expression level when compared to primary cholangiocytes,^14^ providing indirect evidence that GGT levels produced by BCOs might be similar to cholangiocytes. MDR1 activity was assessed by the ability to transfer Rhodamine‐123 into the lumen of the organoids. Upon incubation, Rhodamine‐123 was transported into the organoid lumen (Figure 2D). By blocking the MDR1 channel with MDR1 antagonist verapamil, this luminal accumulation was prevented, confirming the functional MDR1 transporter channel activity in BCOs. The presence of functional cholangiocyte‐specific ion channels in BCOs was analysed on organoids grown on a 2D‐monolayer, using an Ussing chamber setup. Incubation with forskolin (cAMP‐activator) caused an increase in short circuit current (Isc), which could be inhibited by GlyH‐101 (CFTR‐inhibitor). It is important to state that prior to forskolin stimulation, forskolin was still present in the culture medium. Therefore, upon addition, Isc responses (sometimes) can be limited; however, the specific inhibition by GlyH‐101 undoubtedly demonstrated the presence of functional CFTR‐receptors. Incubation with uridine 5'‐triphosphate (UTP) resulted in an increase in Ca^2+^‐dependent channel activation (via purinergic R), which could be inhibited by T16Ainh‐A01 (Figure 2E). These results indicate the presence of functional cholangiocyte ion‐channels CFTR and Anoctamin‐1 (ANO1). All together, these results indicate that BCOs maintain several functional characteristics of cholangiocytes in vitro, but do not represent a fully mature cholangiocyte.

### 3.3 Bile cholangiocyte organoids show similar characteristics as ECOs and ICOs

**FIGURE 3:**
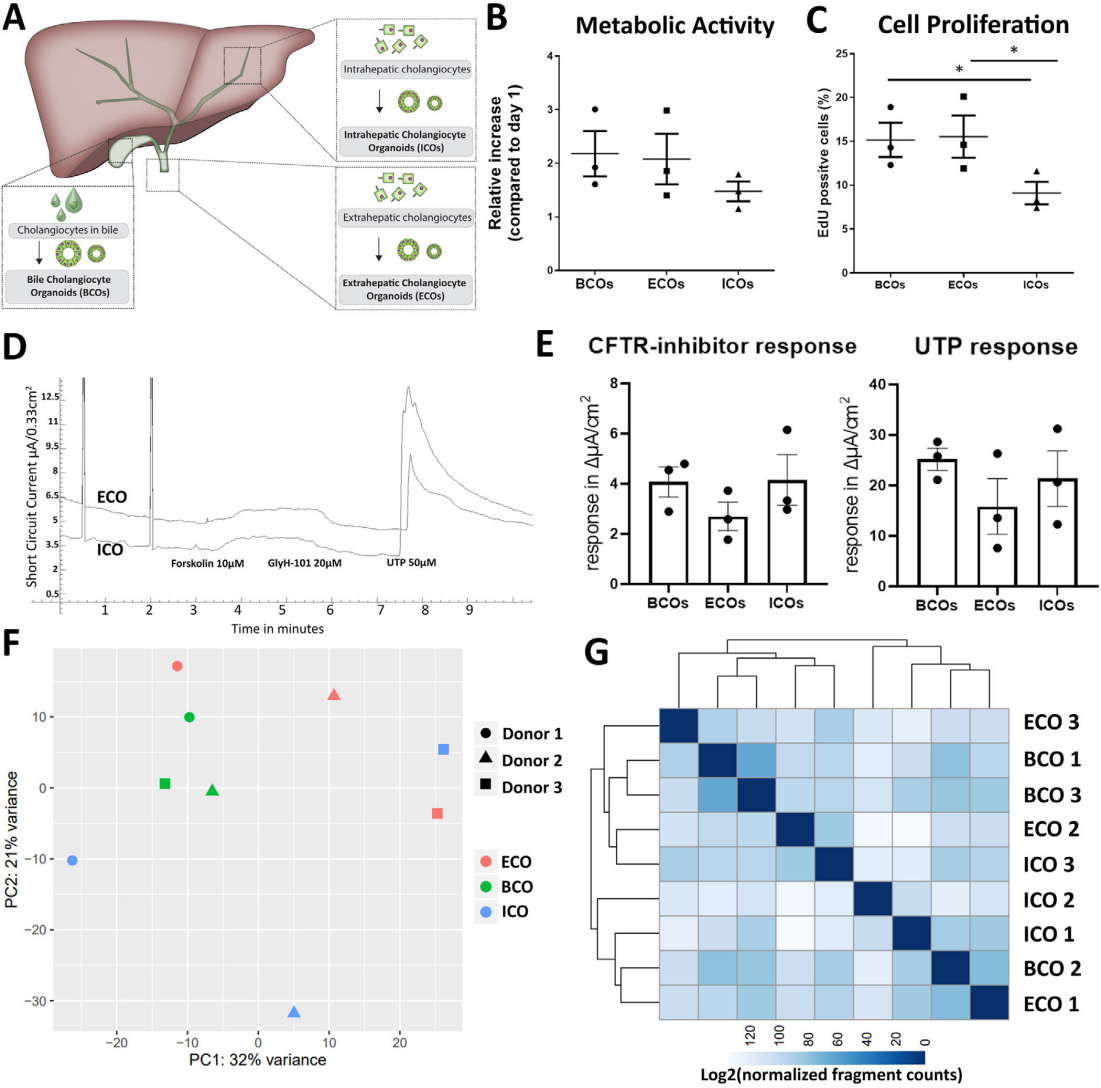
Paired analysis of ICOs, ECOs and BCOs from same donors reveals global similarity and specific differences in cell proliferation. (A) Schematic layout of culture of BCOs, ICOs and ECOs from the same three donors (all experiments performed with donors 1–3). (B) Metabolic activity in the three organoid types as measured by PrestoBlue at day 4 did not increase when compared to day 1 after passaging. (C) Cell proliferation as measured by EdU incorporation. Both BCOs and ECOs have a higher number of EdU‐positive cells compared to ICOs (15.17%±SD3.38 vs. 15.53%±SD4.18 vs. 9.1%±SD2.21, *p* = 0.03). (D) Representative ion‐channel functionality of 2D‐grown ECOs (top line) and ICOs (bottom line) in an Ussing chamber. Stimulation with cAMP‐activator, forskolin (addition to both sides), resulted in an increase in short circuit current. This was completely blocked by CFTR‐inhibitor, GlyH‐101, in addition to the luminal side demonstrating CFTR‐mediated activity. Also, the calcium‐dependent chloride excretion ion‐channel activity (anoctamin‐1) could be specifically stimulated by UTP. (E) Quantification of the Ussing chamber data showed that both CFTR‐activity (as defined by inhibition of the CFTR channel‐activity) and anoctamin‐1‐activity (as defined by UTP stimulation) were similar between cholangiocyte organoids from all three sources. (F) Principal component analysis (PCA), based on top 500 most variable genes determined by RNA‐seq of organoids from all three sources (*n* = 3), showed no clear clustering based upon donor; however, the BCO samples do seem to cluster. (G) Heatmap‐clustering showing sample‐to‐sample distances on the same samples as used in the PCA plot. Scale bar represents the distance between samples. The lower the distance is, the more correlated the samples are. This shows no clear clustering on either source or donor

To investigate how BCOs relate to previously published cholangiocyte organoids derived from tissue,^7^, ^8^ paired organoid cultures were initiated from EHBD and IHBD tissues (ECOs and ICOs, respectively)^28^ and from bile obtained from the gallbladder all from the same patients (*n* = 3, Figure 3A). Morphologically, no obvious differences were observed between the three paired organoid types.^7^, ^8^ The metabolic activity of the three organoid types was analysed using PrestoBlue assay. The increase in metabolic activity over time in BCOs was similar to ECOs (fold change 2.18±SD0.73 vs. 2.08±SD0.82, day 4 activity, relative to day 1) and both had a higher fold change compared to ICOs (fold change 1.47±SD0.32, Figure 3B), although this did not reach statistical significance (*p* = 0.1). We hypothesized that this was caused by lower numbers of proliferating cells in ICOs. However, since the Prestoblue assay only measures broad metabolic activity and not proliferation in particular, we could not rule out that other cellular processes contributed to this difference. Therefore, we quantified the percentage of proliferating cells by flow cytometry analysis of EdU incorporation. As shown in Figure 3C, the EdU‐positive cell subset was similar between BCOs and ECOs (15.17%±SD3.38 vs. 15.53%±SD4.18, *p* = 0.91, Figure 3C), while both had a higher positive subset compared to ICOs (mean 9.1%±SD2.21, *p* = 0.03), confirming our hypothesis. As shown in Figure 3D, ion‐channel functionality of ICOs and ECOs compared to BCOs was assessed. Both ICOs, ECOs and BCOs from the same patients showed similar responses to forskolin and UTP stimulation. When all three organoid pairs were quantified, no significant differences could be found in CFTR‐ or ANO‐1 ion‐channel activity between ICOs, ECOs and BCOs (Figure 3E). These results indicate that the activity of cholangiocyte ion‐channels CFTR and ANO‐1 is comparable between these three organoid types. To identify possible differences between ICOs, ECOs or BCOs, we performed genome‐wide gene expression analyses using bulk RNA‐seq. In Figure 3F, a PCA plot calculated using the top 500 variable genes is shown. Even for the most variable genes, all samples displayed no clear differences to one another and only the BCO samples seem to cluster. When looking at transcriptomic correlation between these samples using heatmap‐clustering for all genes tested (Figure 3G), we do not observe any specific clusters for either source or donor. This indicates that culture conditions, in which (non‐malignant) cholangiocytes are expanded as organoids, are mainly driving the general transcriptomic profile but not the source of the organoids or underlying diseases.

### 3.4 Organoid‐specific and regional gene profiles indicate BCOs are of local (extrahepatic) origin

**FIGURE 4:**
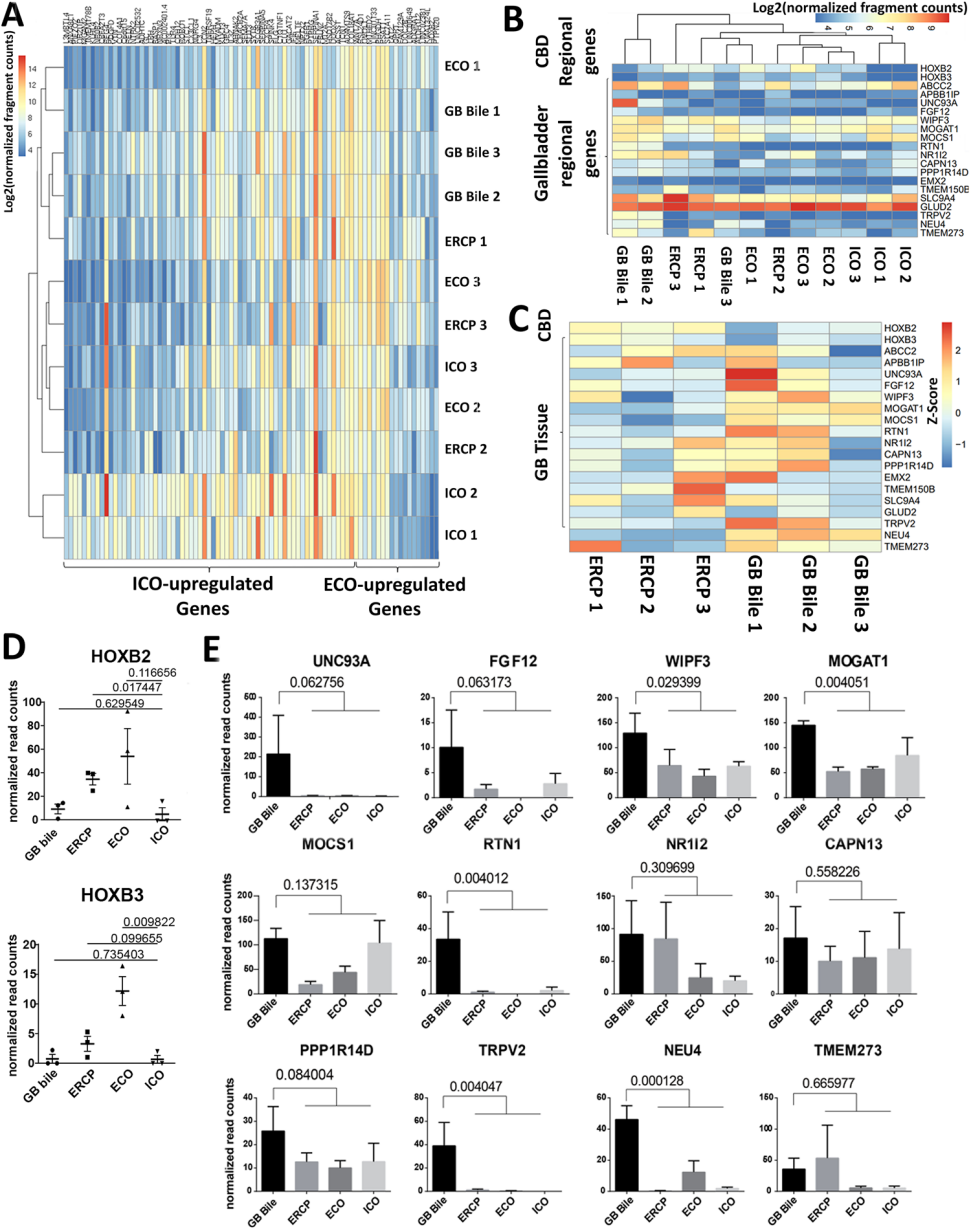
Regional‐specific gene‐expression suggests that BCOs are of local extrahepatic originate. (A) Heatmap of the differentially expressed (DE) genes between ICOs and ECOs (*n* = 3, for detail of genes, Supplementary data file 2), on all organoid samples, showing that ECOs and BCOs cluster more closely. Moreover, BCOs lack the typical ICO‐specific, but do express the ECO‐specific genes. (B) Heatmap of regional‐specific genes preserved in cholangiocyte organoids derived from the common bile duct and gallbladder.^7^ We confirm that ICOs lack expression of *HOXB2* and *HOXB3* as reported.^7^ Both ECOs and ERCP‐derived BCOs express common bile duct genes. Moreover, it was clearly shown that in two (GB bile 1 and 2) out of three samples derived from gallbladder bile, expression of gallbladder tissue–related genes was upregulated compared to other organoids. (C) Heatmap showing Z‐score of the same gene set from B on BCOs from ERCP‐bile and gallbladder‐bile showing the expression of either CBD‐specific or gallbladder tissue‐specific genes, demonstrating regional diversity between BCO sources. (D) Normalized read counts for BCOs (from either GB bile or ERCP), ICOs and ECOs for common bile duct‐specific markers *HOXB2* and *HOXB3* show higher expression in ECOs (for *HOXB3*) and ERCP (*HOXB2*)‐derived samples compared to ICOs. (E) Normalized read counts for BCOs (from either GB bile or ERCP), ICOs and ECOs for 12 GB tissue‐specific genes. Significantly higher expression of 5 of the 12 GB genes was observed in GB‐BCOs as compared to ERCP‐BCOs or ICOs and ECOs. Error bars represent the SEM from three biological replicates. *p*‐values displayed are calculated via *t*‐test and corrected for multiple testing. All experiments in Figure 4 are performed with donors 1–3 and 5–7

One of the major questions considering bile organoids is whether they originate from an intrahepatic location or cholangiocytes of the EHBDs. To investigate this, we compared the transcriptomes of BCOs, both ERCP and gallbladder derived, to ICOs and ECOs. As displayed in Figure S5A and B, PCA of top 500 most variable genes between ERCP‐BCOs and gallbladder BCOs does not result in specific clustering of either type of BCOs towards either ICOs or ECOs. Thus, further analysis was performed focusing on differently expressed (DE) genes between ICOs and ECOs. These ICO‐ and ECO‐unique gene sets are shown in Figure 4A. As shown in Figure 4A and Figure S5C and D, two out of three ICOs had a unique expression profile compared to BCOs and ECOs. One ICO‐line (ICO3) did had absence of ECO‐specific genes, but did not show the unique ICO‐regional gene‐expression (Figure 4A and S5E) that are mostly absent in BCOs from both sources as well (Figure S5E, F), indicating similarities between ECOs and BCOs. Next, we looked at regional gene‐expression profiles that are preserved in cholangiocyte organoids of different parts of the biliary tree tissue as was previously published.^7^ These genes were also specifically analysed in BCOs derived from ERCP and gallbladders. As shown in Figure 4B and C, the typical CBD regional genes *HOXB2* and *HOXB3* ^7^ are highly expressed in ECOs and ERCP‐BCOs, but significantly lower in ICOs and GB‐derived BCOs (Figure 4D). Interestingly, in GB‐derived BCOs, expression of regional‐preserved gallbladder‐specific genes^7^ was more pronounced compared to all other sources of organoids (Figure 4E). Finally, we investigated the transcriptomic difference between ERCP‐derived and GB‐derived BCOs. With this analysis, we could identify 279 DE genes (Supplementary data file 2), indicating that different sources of bile organoids have unique expression profiles and enriched pathways (Figure S6). Overall, these gene‐expression analyses of organoids from different sources indicate that they are highly comparable, but BCOs resemble ECOs more closely when looked at regional‐specific genes. Moreover, different regions of the EHBD are partially preserved in organoids cultured from bile obtained from these regions, showing a high indication that the (majority of) organoid initiating cells come from locally obtained cells; however, some contamination from IHBD cells cannot be excluded.

### 3.5 BCOs and ECOs both lack potential to acquire hepatocyte‐related properties

**FIGURE 5:**
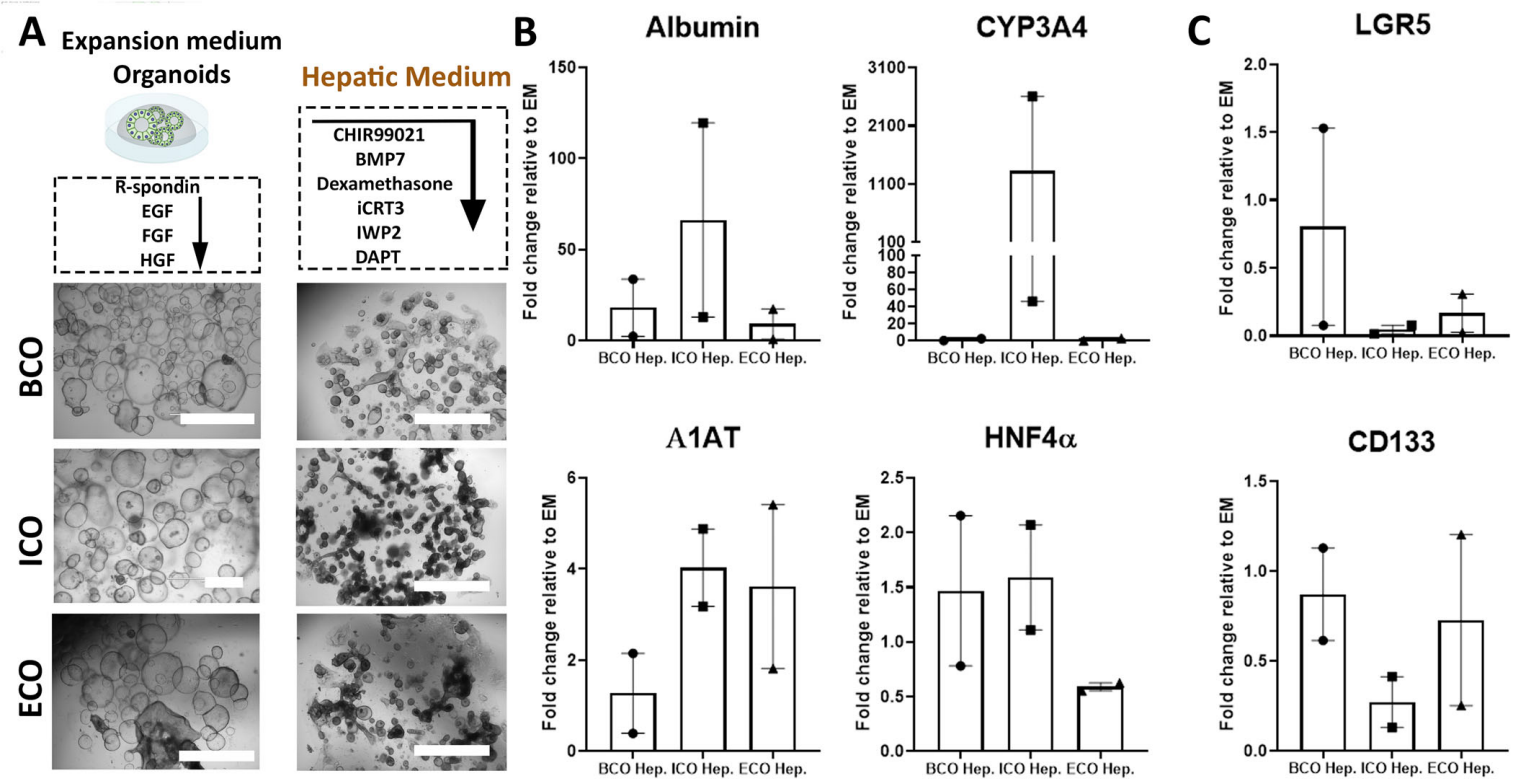
BCOs, similar to ECOs, lack the ability to acquire hepatocyte‐like properties. (A) Schematic overview and pictures of cholangiocyte‐organoids from gallbladder‐derived BCO, ICOs or ECOs (all experiments in Figure 5 are at least performed in three biological replicates with donors 1–4) grown in either in expansion medium containing canonical‐WNT stimulatory factors (left) or after 14 days in hepatic medium (HM, right). Scale bar indicates 2 mm. (B) Gene‐expression by RT‐qPCR for the hepatocyte‐related genes (*Albumin*, *HNF4α*, *CYP3A4* and *Α1AT*) in organoids cultured in HM (*n* = 2). As indicated, only ICOs could upregulate all four hepatocyte‐related genes in individual donors, whereas BCOs and ECOs do not. (C) Gene‐expression by RT‐qPCR relative to the housekeeper gene *HPRT1* for stemness/WNT‐target markers (*LGR5* and *CD133*) from all three sources (*n* = 2) showing the downregulation of stemness/WNT‐target markers for ICOs in HM compared to paired samples in expansion medium

Previous studies showed that ICOs but not ECOs have the potential to upregulate hepatocyte‐specific genes in ‘Hepatic medium (HM)’ conditions (Figure 5A).^7^, ^28^ Since the gene‐expression profiling results indicate that BCOs more closely resemble ECOs, we investigate their ability to acquire hepatocyte‐like potential under these conditions.^7^, ^8^, ^28^, ^29^ To account for inter‐donor variation, we initiated BCOs, ICOs and ECOs from tissue and bile collected from the same patients (*n* = 4). In line with previous studies,^7^, ^27^ big differences in the ability of ICOs to upregulate hepatocyte‐related markers, *Albumin* and *CYP3A4*, in HM conditions were observed between donors. Only two of the four ICO‐lines showed a clear upregulation of these hepatocyte markers (Figure 5B). For the analysis of differentiation of BCOs and ECOs, only the good differentiating donors were included. As shown in Figure 5B, only ICOs and not BCOs or ECOs could upregulate all four hepatocyte markers (*Albumin, CYP3A4, HNF4α* and *Α1AT)* in individual donors when cultured in HM condition. Also, downregulation of stem cell/WNT‐target genes *LGR5* and *CD133* was most profound in ICOs compared to BCOs (Figure 5C). Only one BCO‐line showed 33‐times upregulation of *Albumin* in HM conditions compared to the 120‐times in ICOs from the same donor and is maybe due to contamination of intrahepatic cholangiocytes in this BCO line. When cultured under cholangiocyte‐maturation conditions, all organoids from all sources behaved similar (Figure S7). Overall, our results indicate that none of the ECOs could potentially acquire hepatocytes‐like properties, which is in line with previous results.^7^, ^27^ Moreover, we demonstrate that BCOs have little to no ability to upregulate hepatocyte‐markers as well, confirming that the (bulk of) organoid initiating cells for BCOs are most likely of extrahepatic origin.

### 3.6 BCOs can pave bile ducts scaffolds and acquire tissue‐like cholangiocytes properties in vitro

**FIGURE 6:**
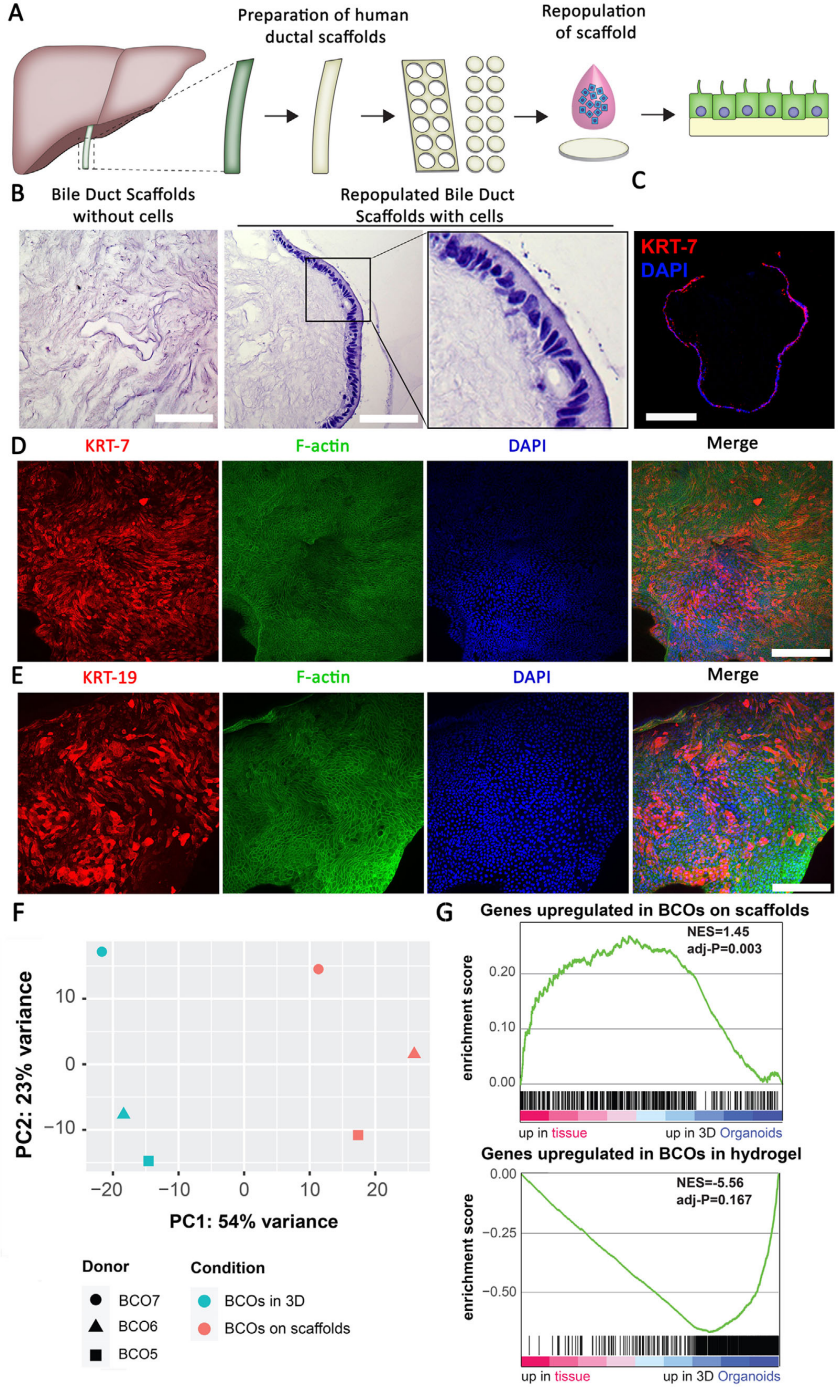
BCOs can pave human extrahepatic bile ducts scaffolds as cholangiocyte‐like cells in vitro. (A) Schematic overview of the experiment. Extrahepatic bile ducts (EHBDs) were removed from their cholangiocyte lining using a Tritox‐X‐100‐based protocol and small punches (circular discs, Ø 3 mm) were created, which were then incubated for 21 days with a BCO cell suspension (consisting of approximately 50·10^3^ single cells). All experiments are performed in triple (both technical and biological, BCO5‐7). (B) H&E confirmed that all cells were efficiently removed from the EHBD scaffolds (left panel) and that single‐cells derived from BCOs repopulate the luminal side of the EHBD scaffolds in a columnar manner (nuclei facing the basolateral side, right panel), scale bars indicate 200 μm (left panel) and 50 μm for the right panel. (C) The recellularized bile duct scaffolds expressed the cholangiocyte marker KRT‐7 (red). Nuclei are stained with DAPI (blue) and scale bar indicates 200 μm. (D) Whole mount confocal imaging of reseeded EHBDs shows nearly complete repopulation of the bile duct lining by BCOs. This included repopulation of the ostia. Most cells expressed cholangiocyte marker KRT‐7 (red), whereas all cells stained positive for Phalloidin (F‐actin, green) and DAPI (nuclei, blue). Scale bars indicate 200 μm. (E) Similar analyses were done by whole mount confocal imaging of cholangiocyte marker KRT‐19, co‐stained with Phalloidin (F‐actin, green) and DAPI (nuclei, blue). A large subset of cells stained positive for KRT‐19 (red) after repopulation. Scale bars indicate 100 μm. (F) PCA plot generated based on RNA‐seq data of BCO samples cultured on scaffolds and in 3D‐hydrogel conditions (*n* = 3 both conditions), showing that the PC1 is mainly determined by the different culture conditions and PC2 mainly by donor. (G) GSEA analysis of differently expressed genes (Supplementary data file 3) between BCOs on scaffolds (upper panel) and BCOs grown in a hydrogel (bottom panel). This gene‐set was compared to the DEgenes from primary cholangiocytes and organoids from the CBD as previously published.^7^ Clearly, BCOs on scaffolds show the highest similarity to primary cholangiocytes of the CBD (*p* = 0.003), while BCOs in hydrogel show a trend towards clustering with the CBD organoid profile

Since most likely BCOs are of extrahepatic origin, we investigated if they could repopulate EHBD scaffold. In Figure 6A, a schematic overview of the experiment is presented. The absence of cells in the EHBD scaffolds after decellularization was confirmed using hematoxylin and eosin (H&E) (Figure 6B, left panel). Repopulation of the EHBD scaffolds with ERCP‐derived BCOs resulted in full coverage of the luminal side of the scaffold surface by a confluent monolayer of cholangiocyte‐like cells after 21 days (Figure 6B, right panel). Cells showed columnar morphology with nuclei on the basolateral side of the cells, while cytoplasm was more prominent at the luminal side, consistent to the histology of bile ducts in vivo (Figure 6B, right panel).^30^ Cross‐sectional imaging of IF staining showed the presence of mature cholangiocyte proteins KRT‐7, KRT‐19, CFTR and SCTR (Figure 6C and S8A). No upregulation of Albumin protein expression was observed (Figure S8A). Whole mount confocal imaging of reseeded EHBDs shows nearly complete paving of the bile duct lining by BCO cells. Furthermore, the ostia located in large bile ducts (peribiliary glands) were repopulated by the cells. All cells stained positive for F‐actin (stained by Phalloidin) and most cells expressed cholangiocyte marker KRT‐7 (Figure 6D and S8B, Video S1) and KRT‐19 (Figure 6E and Video S2).

To gain more insight in cholangiocyte‐like phenotype of the BCO cultured as a monolayer on a human EHBD scaffold, bulk RNA‐seq was performed to look at global gene expression profiles and to compare to those of BCOs cultured in a normal BME hydrogel. As shown in Figure 6F, PCA demonstrates that gene‐expression profiles of BCO cells grown on scaffolds or in a hydrogel display clear differences. Similar differences were also seen using heatmap analyses (Figure S8C). Importantly, 54% of variance in gene expression (PC1) was related to the substrate (scaffold vs. hydrogel), whereas 23% of variance (PC2) was related to the organoid donor (Figure 6F). Overall, when looking in depth at specific cholangiocyte, hepatocyte‐ and cell cycle‐related genes, it is clear that there is no upregulation of hepatocyte‐related genes (Figure S8E, F), but a significant downregulation of *KRT19* upon repopulation of the scaffolds was observed (Figure S8E, F). This is in line with a previous study and our own data of (Figure S4A) that shows that *KRT19* expression in cholangiocyte organoids is higher compared to primary cholangiocytes (Figure S4A).^11^ In addition, the WNT‐target gene *LGR5* and cell cycle‐related genes are severely downregulated in BCOs cultured upon scaffolds (Figure S8D–F), likely as the result of formation of a confluent monolayer and maturation of the cells due to the local environment. Finally, we performed GSEA of BCOs cultured on bile duct scaffolds (Figure 6G, top panel) and in a hydrogel (Figure 6G, bottom panel) comparing the DE genes between primary cholangiocytes and organoids from the CBD that were described by Rimland et al.^7^ It is clear that the unique gene‐expression profile of the repopulated scaffolds shows a significant similarity to that of cholangiocytes from the CBD (*p* = 0.003), while the BCOs cultured in a hydrogel seem to overlap with organoids cultured from the CBD. Collectively, these results provide evidence that cells obtained from bile, and that are expanded as organoids, can efficiently repopulate human bile duct scaffolds without deviation from the biliary lineage and as such obtain gene‐expression profiles more closely resembling primary cholangiocytes in situ.

## 4 DISCUSSION

In this study, we demonstrate that cholangiocyte organoids can be readily cultured from bile and brushes collected *in* and ex vivo. Organoids were cultured from patients and healthy donors, and could be expanded long‐term (>25 weeks, >15 passages) without losing their morphological characteristics using canonical WNT‐stimulating conditions. These BCOs closely resemble ECOs in their gene‐ and protein expression patterns, functionality and inability to differentiate towards hepatocytes. Furthermore, we show that BCOs are capable of repairing damaged human EHBD scaffolds. These findings highlight the potential of BCOs as a patient‐specific minimally invasive cell source for tissue engineering and regenerative medicine applications.

Progress in understanding the pathophysiology and discovering new therapies for biliary diseases has been limited by the lack of functional cholangiocyte‐like cells in vitro. Cholangiocyte organoids, as first described by Huch et al.,^8^, ^31^ have the potential to overcome this problem. Since bile is shown to be a source for cholangiocyte organoids,^13^ it becomes feasible to collect patient samples in a minimally invasive manner during their routine clinical procedures rather than depending on invasive trough‐cut biopsies. Thus, BCOs extend organoid‐initiation beyond patients who receive a liver transplantation or undergo a risky liver biopsy procedure, to patients with rare diseases and following them over time. When cultured in canonical WNT‐stimulating conditions, BCOs show a higher proliferation rate compared to ICOs, making them potentially more suitable for use in large‐scale experiments. Hence, BCOs provide an attractive model to study cholangiocyte biology and (rare) cholangiopathies. Results from our study indicate that organoids obtained from bile (for the majority) have a local origin and retain characteristics of the local bile duct tissue. Two examples of this are the BCOs derived from bile samples from the CBD (by ERCP) and the BCOs derived from gallbladder bile. By comparing the gene expression for organoids with the regional‐specific signature genes that are preserved in organoids identified by Rimland et al.,^7^ we found that ERCP‐derived BCOs upregulated the CBD‐specific genes *HOXB2* and *HOXB3*. Moreover, gallbladder bile‐derived BCOs showed specific upregulation of signature genes expressed in gallbladder‐tissue. Further evidence that BCOs are not derived from IHBDs comes from the fact that they lack the potential to acquire hepatocyte‐like properties. As shown by two previous studies, only ICOs but not ECOs have this potential.^7^, ^28^ This difference in cell fate may be related to their embryogenic origin.^6^ In agreement with these data, we could confirm that only ICOs, but not ECOs from the same patient, are capable of upregulating hepatocyte‐specific genes.^7^, ^28^ In contrast, one BCO line was capable of upregulating *A* *lbumin* expression, although to significantly lower levels compared to the ICO counterpart. Thus, it could be that some intrahepatic cholangiocytes could still end up as organoid‐initiating cells in BCOs. Essentially, our results highlight the resemblance between ECOs and BCOs, suggesting that BCOs should be used to study the EHBD and EHBD‐related cholangiopathies. However, a question that needs to be addressed is why these cholangiocytes are present in bile. It could be due to local turnover or due to mechanical disruption of the epithelium as a result of the procedure. But an alternative explanation would be that these cholangiocytes are harmed by the local disease and as a result detach and end up in the bile. However, this probably would not be harmful for regenerative experiments, since a recent publication showed that cholangiocyte‐organoid initiation likely drives stressed cholangiocytes towards a healthier status and thereby enhancing their regenerative potential after organoid initiation.^27^ Moreover, we demonstrate that there are no large differences between organoids from bile or from healthy donor biopsies. Interestingly, BCOs could allow scientists and clinicians to follow patients during their inevitable disease‐progression. In addition, it would be valuable to research if CCA‐organoids can be cultured from bile. Research indicated that tumour organoids can be cultured from tissue specimens obtained from surgical resections or biopsies, while maintaining their resemblance to the in vivo tumour and can be used for personalized drug screening options.^32^, ^33^, ^34^, ^35^ It is known that CCA cells are detectable in bile.^36^, ^37^, ^38^, ^39^ Thus, it is likely that bile could provide a minimally invasive method to establish CCA organoids, with opportunities to follow CCA development over time. This could accommodate personalized treatment for a larger patient group, especially from patients suffering of CCA in the EHBD, and adjustment of therapy as the tumour progresses and changes.^39^, ^40^

We are not the first group to report on BCOs, Soroka and colleagues showed that BCOs can be cultured from ERCP samples.^13^ In their publication, they provided evidence that some of the inflammatory immune profiles of PSC patients were recapitulated in vitro.^13^ By elaborating on their previous work, we show that BCOs can be formed from multiple sources of bile and from CBD brushes. In line with their results, we demonstrate that BCOs resemble cholangiocyte‐like cells in vitro. In contrast, we failed to show a superiority for ECOs in function and expression of cholangiocyte‐specific channels (CFTR) compared to BCO and ICOs, as was suggested.^13^ Instead, we demonstrate that the transcriptomic profile is highly comparable between organoids from different sources of the same donors (Figure 3G). Soroka et al. accessed expression profiles between BCOs and ICOs from different donors, thus this might explain the discrepancies found between the studies. Moreover, our research focused on the underlying biology of BCOs as well as using them for tissue‐engineering purposes, thus providing a valuable addition to the study by Soroka et al.

We are the first to demonstrate that BCOs cultured in canonical‐WNT conditions can also be used for repopulation of the EHBD by seeding them on empty human scaffolds. These empty scaffolds were created as a model for bile duct damage using a method called decellularization.^26^ We demonstrate that recellularization by BCOs results in the formation of a confluent monolayer with cholangiocyte‐like cells. Importantly, BCOs are less proliferative as well as lose their *LGR5* expression when cultured upon EHBD scaffolds. Furthermore, their gene‐expression profile seems to move towards tissue‐like cholangiocyte as a result of interacting with these scaffolds. Thus, we are the first group that demonstrate the need for appropriate scaffolds for the recreation of EHBD‐constructs. This is in line with previous studies that demonstrated the need for appropriate niches to differentiate and maturate cells.^41^, ^42^, ^43^, ^44^, ^45^ Of note, a recent study indicates that cholangiocytes and cholangiocyte‐organoids are plastic and can adapt their phenotype to local niches in the biliary tree.^27^ As a proof of concept, the authors demonstrate that ECOs can successfully restore IHBDs as intrahepatic cholangiocytes. Similar to their study, after repopulation by BCOs, there was no upregulation of hepatocyte‐related markers.^27^ Moreover, we demonstrate that BCOs are unable to upregulate these hepatocyte‐related makers in vitro. Therefore, it is likely that no other cells from the hepatic‐lineage will be formed when BCOs are used for tissue‐engineering purposes. Additionally, it could be that BCOs can also be used to regenerate the IHBDs in vivo, making BCOs a potential suitable candidate for cholangiopathy treatment.^27^, ^45^

Since we recently showed that our repopulated scaffolds with BCOs are properly polarized by cilia staining and that they become functional constructs by demonstrating transepithelial electrical resistance (TEER) and ion‐channel functionality,^26^ the next goal would be to create a 3D construct. Cholangiocyte‐organoids cultured in non‐canonical WNT‐stimulated conditions have previously shown feasibility to repopulate 3D constructs and function as EHBD in vivo.^14^, ^46^ Thus, it is likely that BCOs can also repopulate 3D constructs. It is important to emphasize that we only decellularized and repopulated the epithelial compartment of the EHBD. For large constructs to function after transplantation, a steady supply of nutrients and oxygen is required. Thus, spontaneous vascularization of the constructs would be necessary. Recent evidence emerged that this might be the case. Both Sampaziotis et al.^14^ and Struecker et al.^47^ showed signs of spontaneous vascularization of EHBD constructs as well as long‐term survival of the animals without biliary complications. Moreover, it was shown that some mesenchymal supportive cells might become spontaneously present as well.^14^ It would have been of great value to see if BCOs can engraft in vivo as well. Although previous studies showed that this is the case for mouse gallbladder‐derived cholangiocyte organoids cultured in canonical WNT‐stimulating conditions,^48^ there is still a need to demonstrate this for human BCOs as well. Our laboratory lacks the animal micro‐surgery expertise and medical ethical approval to perform bile duct transplantations in mice, but in collaboration with expert labs, these can hopefully be performed in the future. Furthermore, clinical applications of organoids are still (partially) limited by the use of non‐GMP‐compliant mouse tumour ECM extracts, such as matrigel and BME. Recent studies have shown that organoids can be cultured in more clinically relevant hydrogels derived from porcine small intestine submocase^49^ or cellulose nanofibril.^50^ Thus, in theory, patient‐derived BCOs cultured in these ECM hydrogels could effectively create EHBDs in vitro, which might be transplanted back into the patient of which the bile is obtained.

In conclusion, our study shows that bile obtained from multiple sources from a wide range of patients can be used to culture cholangiocyte organoids from the EHBD. This opens new doors to study extrahepatic biliary diseases and regenerative medicine of the extrahepatic bile duct without the need of invasively collected biopsies.

## FUNDING

This project was partially funded by the MLDS‐Diagnostiek grant D16‐26 of the Dutch Digestive Disease Foundation and an Erasmus MC ‘PhD Project’ Grant.

## DISCLOSURES

On behalf of all authors, I hereby confirm that there are no conflicts of interest.

## Supporting information

SUPPORTING INFORMATION

SUPPLEMENTAL VIDEO 1

SUPPLEMENTAL VIDEO 2

## References

[1] Tabibian JH , Masyuk AI , Masyuk TV , et al. Physiology of cholangiocytes. Compr Physiol. 2013;3:1‐49. 23720296 10.1002/cphy.c120019 PMC3831353

[2] Zong Y , Stanger BZ . Molecular mechanisms of bile duct development. Int J Biochem Cell Biol. 2011;43:257–264. 20601079 10.1016/j.biocel.2010.06.020 PMC2990791

[3] Banales JM , Huebert RC , Karlsen T , et al. Cholangiocyte pathobiology. Nat Rev Gastroenterol Hepatol. 2019;16(5):269–281. 30850822 10.1038/s41575-019-0125-y PMC6563606

[4] Karimian N , Op den Dries S , Porte RJ . The origin of biliary strictures after liver transplantation: is it the amount of epithelial injury or insufficient regeneration that counts? J Hepatol. 2013;58:1065–1067. 23466306 10.1016/j.jhep.2013.02.023

[5] Chapman R , Cullen S . Etiopathogenesis of primary sclerosing cholangitis. World J Gastroenterol. 2008;14:3350–3359. 18528932 10.3748/wjg.14.3350 PMC2716589

[6] Fabris L , Fiorotto R , Spirli C , et al. Pathobiology of inherited biliary diseases: a roadmap to understand acquired liver diseases. Nat Rev Gastroenterol Hepatol. 2019;16(8):497–511. 31165788 10.1038/s41575-019-0156-4 PMC6661007

[7] Rimland CA , Tilson SG , Morell CM , et al. Regional differences in human biliary tissues and corresponding in vitro derived organoids. Hepatology. 2021;73(1):247–267. 32222998 10.1002/hep.31252 PMC8641381

[8] Huch M , Gehart H , van Boxtel R , et al. Long‐term culture of genome‐stable bipotent stem cells from adult human liver. Cell. 2015 Jan 15;160(1‐2):299–312, 10.1016/j.cell.2014.11.050. 25533785 PMC4313365

[9] Planas‐Paz L , Sun T , Pikiolek M , et al. YAP, but not RSPO‐LGR4/5, signaling in biliary epithelial cells promotes a ductular reaction in response to liver injury. Cell Stem Cell. 2019;25(1):39–53.e10. 31080135 10.1016/j.stem.2019.04.005

[10] Pepe‐Mooney BJ , Dill MT , Alemany A , et al. Single‐cell analysis of the liver epithelium reveals dynamic heterogeneity and an essential role for YAP in homeostasis and regeneration. Cell Stem Cell. 2019;25(1):23–38.e8. 31080134 10.1016/j.stem.2019.04.004 PMC6814390

[11] Aizarani N , Saviano A , Sagar , et al. A human liver cell atlas reveals heterogeneity and epithelial progenitors. Nature. 2019;572(7768):199–204. 31292543 10.1038/s41586-019-1373-2 PMC6687507

[12] Aloia L , McKie MA , Vernaz G , et al. Epigenetic remodelling licences adult cholangiocytes for organoid formation and liver regeneration. Nat Cell Biol. 2019;21(11):1321–1333. 31685987 10.1038/s41556-019-0402-6 PMC6940196

[13] Soroka CJ , Assis DN , Alrabadi LS , et al. Bile‐derived organoids from patients with primary sclerosing cholangitis recapitulate their inflammatory immune profile. Hepatology. 2019;70(3):871‐882. 30561836 10.1002/hep.30470

[14] Sampaziotis F , Justin AW , Tysoe OC , et al. Reconstruction of the mouse extrahepatic biliary tree using primary human extrahepatic cholangiocyte organoids. Nat Med. 2017;23:954–963. 28671689 10.1038/nm.4360

[15] Roest HP , Ooms LSS , Gillis AJM , et al. Cell‐free microRNA miR‐505‐3p in graft preservation fluid is an independent predictor of delayed graft function after kidney transplantation. Transplantation. 2019;103(2):329‐335. 30444806 10.1097/TP.0000000000002527

[16] Sampaziotis F , de Brito MC , Madrigal P , et al. Cholangiocytes derived from human induced pluripotent stem cells for disease modeling and drug validation. Nat Biotechnol. 2015;33(8):845‐852. 26167629 10.1038/nbt.3275 PMC4768345

[17] Dobin A , Davis CA , Schlesinger F , et al. STAR: ultrafast universal RNA‐seq aligner. Bioinformatics. 2013;29(1):15‐21. 23104886 10.1093/bioinformatics/bts635 PMC3530905

[18] Li H , Handsaker B , Wysoker A , et al. The Sequence Alignment/Map format and SAMtools. Bioinformatics. 2009;25(16):2078‐2079. 19505943 10.1093/bioinformatics/btp352 PMC2723002

[19] Liao Y , Smyth GK , Shi W . featureCounts: an efficient general purpose program for assigning sequence reads to genomic features. Bioinformatics. 2014;30(7):923‐930. 24227677 10.1093/bioinformatics/btt656

[20] Love MI , Huber W , Anders S . Moderated estimation of fold change and dispersion for RNA‐seq data with DESeq2. Genome Biol. 2014;15(12):550. 25516281 10.1186/s13059-014-0550-8 PMC4302049

[21] Kuleshov MV , Jones MR , Rouillard AD , et al. Enrichr: a comprehensive gene set enrichment analysis web server 2016 update. Nucleic Acids Res. 2016;44(W1):W90–W97. 27141961 10.1093/nar/gkw377 PMC4987924

[22] Schneeberger K , Sánchez‐Romero N , Ye S , et al. Large‐scale production of LGR5‐positive bipotential human liver stem cells. Hepatology. 2020;72(1):257‐270. 31715015 10.1002/hep.31037 PMC7496924

[23] Korotkevich , G , Sukhov V & Sergushichev A Fast gene set enrichment analysis. 2019. bioRxiv 060012; 10.1101/060012.

[24] Stuart T , Butler A , Hoffman P , et al. Comprehensive integration of single‐cell data. Cell. 2019;177(7):1888‐1902.e21. 31178118 10.1016/j.cell.2019.05.031 PMC6687398

[25] Clevers H , Gehart H . WO2017149025 — improved differentiation method. Available at: https://patentscope.wipo.int/search/en/detail.jsf?docId=WO2017149025&tab=PCTBIBLIO Accessed on May 1, 2020.

[26] Willemse J , Roos FJM , Voogt IJ , et al. Scaffolds obtained from decellularized human extrahepatic bile ducts support organoids to establish functional biliary tissue in a dish. Biotechnol Bioeng. 2021;118(2):836‐851. 33118611 10.1002/bit.27613 PMC7894321

[27] Sampaziotis F , Muraro D , Tysoe OC , et al. Cholangiocyte organoids can repair bile ducts after transplantation in the human liver. Science. 2021;371(6531):839‐846. 33602855 10.1126/science.aaz6964 PMC7610478

[28] Verstegen MMA , Roos FJM , Burka K , et al. Human extrahepatic and intrahepatic cholangiocyte organoids show region‐specific differentiation potential and model cystic fibrosis‐related bile duct disease. Sci Rep. 2020;10(1):21900 33318612 10.1038/s41598-020-79082-8 PMC7736890

[29] Raven A , Lu WY , Man TY , et al. Cholangiocytes act as facultative liver stem cells during impaired hepatocyte regeneration. Nature. 2017;547(7663):350‐354. 28700576 10.1038/nature23015 PMC5522613

[30] Boyer JL . Bile formation and secretion. Compr Physiol. 2013;3(3):1035–1078 23897680 10.1002/cphy.c120027 PMC4091928

[31] Broutier L , Andersson‐Rolf A , Hindley CJ , et al. Culture and establishment of self‐renewing human and mouse adult liver and pancreas 3D organoids and their genetic manipulation. Nat Protoc. 2016;11:1724–1743. 27560176 10.1038/nprot.2016.097

[32] Broutier L , Mastrogiovanni G , Verstegen MMA , et al. Human primary liver cancer‐derived organoid cultures for disease modeling and drug screening. Nat Med. 2017;23(12):1424‐1435. 29131160 10.1038/nm.4438 PMC5722201

[33] Nuciforo S , Fofana I , Matter MS , et al. Organoid models of human liver cancers derived from tumor needle biopsies. Cell Rep. 2018;24(5):1363–1376. 30067989 10.1016/j.celrep.2018.07.001 PMC6088153

[34] Saito Y , Muramatsu T , Kanai Y , et al. Establishment of patient‐derived organoids and drug screening for biliary tract carcinoma. Cell Rep. 2019;27:1265–1276. 31018139 10.1016/j.celrep.2019.03.088

[35] Kopper O , de Witte CJ , Lõhmussaar K , et al. An organoid platform for ovarian cancer captures intra‐ and interpatient heterogeneity. Nat Med. 2019;25(5):838‐849. 31011202 10.1038/s41591-019-0422-6

[36] Macias RIR , Banales JM , Sangro B , et al. The search for novel diagnostic and prognostic biomarkers in cholangiocarcinoma. Biochim Biophys Acta Mol Basis Dis. 2018;1864(4 Pt B):1468‐1477. 28782657 10.1016/j.bbadis.2017.08.002

[37] Shen N , Zhang D , Yin L , et al. Bile cell‐free DNA as a novel and powerful liquid biopsy for detecting somatic variants in biliary tract cancer. Oncol Rep. 2019;42(2):549‐560. 31173267 10.3892/or.2019.7177 PMC6610033

[38] Lee SJ , Lee YS , Lee MG , et al. Triple‐tissue sampling during endoscopic retrograde cholangiopancreatography increases the overall diagnostic sensitivity for cholangiocarcinoma. Gut Liver. 2014;8(6):669‐673. 25368755 10.5009/gnl13292 PMC4215455

[39] Razumilava N , Gores GJ . Cholangiocarcinoma. Lancet. 2014;383(9935):2168‐2179. 24581682 10.1016/S0140-6736(13)61903-0 PMC4069226

[40] Rizvi S , Gores GJ . Pathogenesis, diagnosis, and management of cholangiocarcinoma. Gastroenterology. 2013;145(6):1215‐1229. 24140396 10.1053/j.gastro.2013.10.013 PMC3862291

[41] Badylak SF , Freytes DO , Gilbert TW . Extracellular matrix as a biological scaffold material: structure and function. Acta Biomater. 2009;5(1):1‐13. 18938117 10.1016/j.actbio.2008.09.013

[42] Gjorevski N , Sachs N , Manfrin A , et al. Designer matrices for intestinal stem cell and organoid culture. Nature. 2016;539(7630):560‐564. 27851739 10.1038/nature20168

[43] Nikolaev M , Mitrofanova O , Broguiere N , et al. Homeostatic mini‐intestines through scaffold‐guided organoid morphogenesis. Nature. 2020;585(7826):574‐578. 32939089 10.1038/s41586-020-2724-8

[44] Lorvellec M , Scottoni F , Crowley C , et al. Mouse decellularised liver scaffold improves human embryonic and induced pluripotent stem cells differentiation into hepatocyte‐like cells. PLoS One. 2017;12(12):e0189586. 29261712 10.1371/journal.pone.0189586 PMC5738056

[45] Willemse J , Lieshout R , van der Laan LJW , et al. From organoids to organs: bioengineering liver grafts from hepatic stem cells and matrix. Best Pract Res Clin Gastroenterol. 2017;31(2):151‐159. 28624103 10.1016/j.bpg.2017.03.003

[46] Tysoe OC , Justin AW , Brevini T , et al. Isolation and propagation of primary human cholangiocyte organoids for the generation of bioengineered biliary tissue. Nat Protoc. 2019;14(6):1884‐1925. 31110298 10.1038/s41596-019-0168-0

[47] Struecker B , Hillebrandt KH , Raschzok N , et al. Implantation of a tissue‐engineered neo‐bile duct in domestic pigs. Eur Surg Res. 2016;56(1‐2):61‐75. 26684913 10.1159/000441720

[48] Lugli N , Kamileri I , Keogh A , et al. R‐spondin 1 and noggin facilitate expansion of resident stem cells from non‐damaged gallbladders. EMBO Rep. 2016;17(5):769‐779. 26993089 10.15252/embr.201642169 PMC5341509

[49] Giobbe GG , Crowley C , Luni C , et al. Extracellular matrix hydrogel derived from decellularized tissues enables endodermal organoid culture. Nat Commun. 2019;10(1):5658. 31827102 10.1038/s41467-019-13605-4 PMC6906306

[50] Krüger M , Oosterhoff LA , van Wolferen ME , et al. Cellulose nanofibril hydrogel promotes hepatic differentiation of human liver organoids. Adv Healthcare Mater. 2020;9(6):e1901658. 10.1002/adhm.201901658 32090504

