# Supplementary material for: Cholangiocyte organoids from human bile retain a local phenotype and can repopulate bile ducts in vitro
Source: Clin Transl Med. 2021 Dec 26;11(12):e566. doi: 10.1002/ctm2.566 (PMC8710298; doi:10.1002/ctm2.566)
Supplement: Supplementary file 1 — SUPPORTING INFORMATION [file CTM2-11-e566-s001.docx]

**Supplementary Information

Materials and Methods

Tissue collection**Biopsies were stored at 4˚C in University of Wisconsin (UW, Bridge to Life Ltd. Belzer Cold Storage Solution) preservation solution during transport. For a complete overview of characteristics of patients or donors from whom (liquid) biopsies were obtained and successfully cultured as organoids, see Table 1 and Table S1. All patients or their next of kin gave written informed consent to use their tissue collected during liver transplantation for research purposes. The use of tissue biopsies and livers deemed unsuitable for transplantation was approved by the Medical Ethical Committee (MEC) of the Erasmus MC, (MEC-2012-090, MEC-2014-060).

**Initiation and culture expansion of tissue-derived organoids**
Biopsies were digested by incubation with 4 mL collagenase digesting solution (2.5 mg/mL collagenase A1, Roche) in Earle's Balanced Salt Solution (EBSS, Hyclone, Thermoscientific) for 30 min at 37 ˚C. Digestion solution was diluted by adding cold Advanced (Adv)DMEM/F12 (GIBCO, supplemented with 100µg/mL penicillin/streptomycin, Life Technologies; HEPES 1M, Fisher Scientific; 1% Ultraglutamine 200mM, Fisher Scientific and 100µg/mL Primocin, Invivogen) and centrifuged for 5 minutes, 4˚C at 453g. The cell suspension was filtered through a 70 µm Nylon cell strainer and centrifuged for 5 minutes, 4˚C at 453g. Supernatant was removed and the cell pellet was suspended in cold (4°C) 25µL matrigel (Corning Incorporation) or 25µL Basement Membrane Extract (BME, Cultrex) diluted with 30% of AdvDMEM/F12 which was allowed to solidify for 30-45 minutes at 37°C before 250µL start-up expansion medium (SEM, table S2)^1^ was added. SEM was replaced with canonical-WNT stimulating expansion medium (EM, table S2) after three days.^1^ In addition to extrahepatic cholangiocyte organoids (ECOs) cultured in canonical-WNT conditions (table S2), ECOs were created from gallbladder tissue biopsies cultured in non-canonical WNT-stimulation conditions as a positive control for the γ-glutamyltranspeptidase assay. These organoids were created in a similar manner as described above, only cultured in the non-canonical WNT stimulating medium as published by Sampaziotis *et al.* (n=3).^2^
**Initiation and culture expansion of bile-derived organoids**
In short, bile was washed twice in 8 mL cold AdvDMEM/F12 and centrifugation for 5 minutes at 4°C at 453g. Subsequently, the supernatant was removed, the cell pellet was suspended in 3 mL of AdvDMEM/F12, and filtered through a 100 µm cell strainer to get a single cell suspension. After a third wash with 3 mL of AdvDMEM/F12, the cell pellet was collected and seeded either in 25µL matrigel (Corning Incorporation) or 70% BME (Cultrex) diluted with AdvDMEM/F12. SEM (250µL) was added for the first three days and then changed to EM according to the previously published protocol and table S2.^1,3^
If bile was collected via ERCP or PTCD from patients having symptoms of bacterial cholangitis (either jaundice, fever or severe stomach ache), the medium was supplemented with vancomycin, (50µg/mL) during the first three days of culture. The effect of this treatment on organoid culture was evaluated in a dose dependent manner using AlamarBlue (ThermoFisher) assay, performed according to the manufacturer’s instructions, and showed no effect on cell viability (Figure S1).
Cultures initiated from PSC or cholangiocarcinoma (CCA) patient bile were supplemented with 1% Antibiotic-Antimycotic (Gibco), instead of vancomycin, during the first three days as published previously^4^ to minimalize the risk of fungal infection. EM was refreshed every 3 or 4 days on all cultures and they were passaged in a 1:2-1:8 ratio according to proliferation rate. All organoid cultures described were tested and found negative for mycoplasma (data not shown).
 **Time window for efficient organoid initiation**To evaluate the optimal timeframe to process bile after collection, ERCP-obtained bile was divided into two aliquots of 1 mL. One aliquot was processed immediately after collection (< 1 hour), the other sample was stored for four hours at 4˚C before organoid culture initiation. After 7 days of culture, the numbers of organoids larger than 100 µm in diameter were assessed (Invitrogen™ EVOS™ FL Digital Inverted Fluorescence Microscope). **Immunofluorescence (IF)** **staining**Organoids were fixed for 10 minutes using 4% paraformaldehyde removing the hydrogel. Samples were paraffin embedded and cut as 4 µm thick slides. Next, they were permeabilized with 0.1% Triton X-100 diluted in PBS for 15 minutes. Subsequently, they were exposed to 10% serum diluted in 1% BSA-PBS to prevent a-specific antibody binding. Primary antibodies were added to the organoids and incubated overnight at 4˚C. Finally, incubation with the secondary antibody (Table S4) took place for 60 minutes at room temperature and cell nuclei were stained with DAPI (Vectashield, Vectorlabs) and analysis took place on a SP5 confocal microscope (LEICA) equipped with a 405, 488 and 561 nm laser. Images were analysed using ImageJ (version 1.52p, supplemented with FIJI).

**Ussing chamber assay**Prior to seeding of the cells, transwell inserts were coated with 5% matrigel in PBS for 2 hours. Fully expanded domes of organoids (20 fully grown domes of 25µL) were collected in AdvDMEM/F12 and centrifuged (453*g*, 5 min, 4˚C). After removal of the supernatant, organoids were mechanically broken by vigorously up and down pipetting. The organoid suspension was spun down again and the cell pellet was made single cell by digestion in Trypsin-EDTA (TE) for 25 to 40 min at 37°C. Cells were washed in AdvDMEM/F12 and sieved through a cell 70 µm cell strainer. Approximately 3x10^5^ cells were suspended in 200 µL EM and seeded on transwell inserts. Medium was changed every 3-4 days. To check confluency, the cells were examined by daily microscopy and electrophysiological analysis was performed after 4 days. Upon forming a confluence monolayer, transwells were placed in an Ussing chamber (Physiologic instruments) set up to analyze functional cholangiocyte-specific transporter channels (Cystic Fibrosis Transmembrane Conductance Regulator –CFTR- and Ca^2+^- activated Cl^-^ channel) using Acquire & Analyze Software 2.3 (Physiologic Instruments, San Diego, California). The temperature of the chambers was kept at 37°C by warm water bath circulation and chambers were gassed with 95%O_2_, 5%CO_2_. Each chamber consisted of 3mL modified Meyler solution (128 mmol/liter NaCl, 4.7 mmol/liter KCl, 1.3 mmol/liter CaCl_2_, 1.0 mmol/liter MgCl_2_, 0.3 mmol/liter Na_2_HPO_4_, 0.4 mmol/liter NaH_2_PO_4_, 20 mmol/liter NaHCO_3_, 10 mmol/liter HEPES, supplemented with glucose (10 mmol/liter) at pH 7.3). Current was clamped and every second short circuit current (Isc) was recorded. CFTR-dependent anion secretion was activated by adding Forskolin (3μL, 10mM) to both sides of the cells, and GlyH-101 (3μL, 20mM, apical). Calcium (Ca^2+^) activated chloride (Cl^-^) channels (CaCC) were stimulated by UTP (3μL, 50mM, apical) and inhibited by T16Ainhibitor-A01 (3-5 μL, 50mM, apical). The Isc measurements are presented as measured (μA/0.33cm^2^).
**Metabolic activity measurement**Metabolic activity in organoids from bile and intra- and extrahepatic bile duct biopsies (n=3 all sources) was determined using the PrestoBlue metabolic assay. In short, 400 µL of diluted PrestoBlue (10% in AdvDMEM/F12) was added per 25 µL dome (48 well plate culture suspension, Corning). After 4 hours of 37˚C incubation, 100 µL of PrestoBlue solution per dome was transferred to a white walled 96 well plate (Perkin Elmer) and fluorescence intensity was measured using a plate reader (CytoFluor Series 4000, Applied Biosystems) with the excitation/emission wavelengths set to 530/590nm. BME without cells was measured to assess the background fluorescent signal. This measurement was repeated after three days of subsequent culture and the relative increase in PrestoBlue per dome was calculated.

**Cell proliferation**
EdU-incorporation was performed according to the manufacturer’s protocol. In short: EdU (10µM) was added to the medium and incubated for 4 hours at 37˚C degrees. The organoids were dissociated into single cell suspension as described earlier and suspended in 200µL of 1%BSA-PBS to be analysed by flow cytometry (Canto flow cytometer, BD Biosciences). The gating strategy is shown in Figure S3. Subset analysis was done using Flowjo (version v10.6.1, BD) analysis software.
 **Upregulation of hepatocyte-specific markers/hepatocyte (trans)differentiation**In principle, upregulation of hepatocyte-specific markers/hepatocyte (trans)differentiation by the novel defined culture conditions is based upon removal of WNT/β-catenin stimulators and blocking of notch-related cholangiocyte differentiation. To achieve upregulation of hepatocyte-associated markers. Organoids were passaged once at 7 days during differentiation and the total differentiation protocol took 14 days (including pre-treatment with BMP7). Differentiation was confirmed by gene and protein expression analysis of genes associated with hepatocyte maturation (*Albumin*, *HNF4α, CYP3A4* and *alpha-1-anti trypsin* –*A1AT*-*)*^5^ and stemness/WNT-target genes (*LGR5* and *CD133*)^5^ by RT-qPCR. These data are presented in fold change manner comparing them to their expansion medium controls.
 **Cholangiocyte maturation**
Cholangiocyte-maturation medium for cholangiocyte organoids (COs) was based upon the induced-pluripotent stem cell (IPS) protocol for cholangiocyte-differentiation as established by Sampaziotis *et al.*^5,6^ and adapted by Verstegen MMA *et al.*^7^ for tissue-derived cholangiocyte organoid cultures. This two-step protocol largely followed the principles of differentiation hepatoblast-like IPS towards cholangiocytes. Bile-cholangiocyte organoids (BCOs), ECOs and Intrahepatic cholangiocyte organoids (ICOs) from three donors were exposed to these culture conditions and compared to their expansion culture conditions.^1^ In detail, all cholangiocyte organoids were expanded for 7 days in basement membrane extract diluted in 10% Williams-E medium (WE, Gibco, Life Technologies) to near-full wells. Culture medium was then switched to maturation medium, consisting of AdvDMEM/F12 supplemented with 1:50 B27 (Gibco), 50 ng/mL FGF10 (Peprotech), 50 ng/mL Activin-A (Gibco) and 3 μM retinoic acid (Sigma-Aldrich). After 4 days, medium was changed to WE supplemented with 10 mM nicotinamide (Sigma-Aldrich), 17 mM sodium bicarbonate (Sigma-Aldrich), 0.2 mM 2-phospho-i-ascorbic acid tri-sodium salt (Sigma-Aldrich), 6.3 mM sodium pyruvate (Invitrogen), 14 mM glucose (Sigma-Aldrich), 20 mM HEPES (Fisher Scientific), ITS+ premix (BD Biosciences), 0.1 M dexamethasone (R&D), 20 ng/mL EGF (R&D), 2 mM Ultraglutamine (Invitrogen), and penicillin (100 U/mL) & streptomycin (100g/mL). The medium was refreshed every 2 days for a total of 10 days after which the organoids were analysed. Maturation *in vitro* was assessed by gene-expression profiles using RT-qPCR and immunofluorescence for protein-expression of mature cholangiocyte markers (cytokeratin –*KRT-7*, *KRT19*, *AQP1* and *CFTR*) as well as looking at progenitor/stem cell/WNT-target markers (*LGR5* and *SOX9*).^6^

**Repopulation of EHBD scaffolds**
EHBD scaffolds were prepared as previously published.^8^ After procurement of donor livers deemed unsuitable for transplantation, EHBD (n=3, length: 4cm) were surgically removed, stored in 0.9% saline solution and frozen at -20°C. Decellularisation was started after complete thawing of EHBD tissue. Subsequently, cells were removed by submersion in 4% Triton-X-100 + 1NH_3_. The Triton solution was refreshed every 30 minutes for a total of 10 cycles. Afterwards, the EHBD segments were washed with dH_2_O until all detergent was removed. This was followed by DNase type1 treatment in 0.9% NaCl + 100mM CaCl_2_ + 100mM MgCl_2_ treatment. Complete decellularisation was confirmed with hematoxylin and eosin (H&E) staining. H&E stained slides were imaged with Zeiss Axiokop 20 microscope and captured with a Nikon DS-U1 camera. EHBD scaffolds were prepared using a dermal biopsy punch (Ø 3mm). These discs were reseeded by incubation with single cell suspensions made from BCOs. This cell suspension (5.0·10^3^ cells/µL, 10 µL) was pipetted on top of the ductal scaffolds and incubated for 4 hours at 37°C to settle. After this, 500 µL EM supplemented with 10 µM Y27632 was added. After 3 days, the medium was changed with 500 µL EM without Y27632. Medium was refreshed every 2-3 days. Reseeded scaffolds were cultured for 21 days and subsequently fixed in 4% paraformaldehyde for 20 minutes. Subsequently, immunofluorescence staining with the primary antibodies, KRT-7, SCTR, CFTR and Albumin was performed on sections as previously described under the immunofluorescence section in the methods. Furthermore, repopulation efficacy was determined by whole mount confocal microscopy to assess KRT-7 and KRT-19 protein expression. For this, reseeded EHBD samples were stained with fluorescently labeled antibodies in a similar manner as described earlier. Additional cytoskeletal staining with Phalloidin Alexa Fluor™ 488 (1:200, ThermoFisher) and nuclear staining with DAPI was performed. Samples were imaged using a Leica 20X water dipping lens on Leica DM6000 CFS microscope with a LEICA TCS SP5 II confocal system. Images were processed using ImageJ.

**Tables and Figures**

**Table S1. Characteristics of additional *in vivo* collected bile and brush samples with successful organoid initiation**

| **Age patient (years)** | **Sex** | **Bile Source** | **Donor Type or Indication ERCP/surgery** |
| --- | --- | --- | --- |
| 48 | M | ERCP | AS |
| 63 | M | ERCP | AS |
| 66 | M | ERCP | Mirizzi Syndrome |
| 31 | M | Brush | PSC |
| 41 | M | ERCP | AS |
| 77 | F | PTCD | CCA |
| 71 | M | ERCP | NAS |
| 64 | M | ERCP | AS |
| 26 | F | ERCP | AS |
| 57 | F | ERCP | AS |
| 66 | M | ERCP | AS |
| 73 | M | PTCD | NAS |
| 79 | F | ERCP | CCA |
| 60 | M | ERCP | AS |
| 20 | M | ERCP | PSC |
| 66 | F | ERCP | CCA |
| 49 | M | ERCP | AS |
| 68 | M | ERCP | AS |
| 66 | F | ERCP | CCA |
| 48 | M | ERCP | AS |
| 52 | M | ERCP and brush | CCA |
| 58 | M | ERCP | AS |
| 57 | F | ERCP | AS |
| 57 | F | PTCD | CCA |
| 71 | M | ERCP | NAS |
| 55 | M | ERCP | PSC |
| 71 | M | ERCP | Bile Stones |
| 63 | M | ERCP | CCA |
| 73 | F | ERCP | Bile Stones |
| 61 | M | ERCP | AS |
| 59 | F | ERCP | PSC |
| 26 | F | ERCP | Bile Stones |
| 58 | F | ERCP | AS |
| 70 | M | ERCP | Bile Stones |
| 72 | M | ERCP | Bile Stones |
| 20 | M | ERCP | AS |
| 54 | F | ERCP | AS |
| 54 | M | ERCP | Bile Stones |
| 63 | M | ERCP | AS |
| 85 | M | ERCP | Bile Stones |
| 50 | M | ERCP | AS |
| 54 | F | ERCP | Papiladenoma |
| 30 | M | ERCP | PSC |

**Abbreviations:** AS: Anastomotic bile duct Stricture, CCA: Cholangiocarcinoma, ERCP: Endoscopic Retrograde Cholangiopancreaticography, F: Female, M: Male, NAS: Non-Anastomotic bile duct Stricture, PSC: Primary Sclerosing Cholangitis, PTCD: Percutaneous Transhepatic Cholangiography Drainage.

**Table S2. Culture conditions used within this manuscript.**

**Medium formulation for Start Up Medium (SEM) and Expansion Medium (EM) per mL. Medium components with a * are only added to SEM.**

| **Component** | **Concentration** | **Brand** |
| --- | --- | --- |
| Supplemented AdvDMEM/F12** |  | Gibco |
| N2 | 1% | Gibco |
| B27 | 2% | Gibco |
| N-Acetylcystein | 1,25 mM | Sigma |
| gastrin | 10 nM | Sigma |
| EGF | 50 ng/mL | Peprotech |
| FGF10 | 100 ng/mL | Peprotech |
| HGF | 25 ng/mL | Peprotech |
| nicotinamide | 10nM | Sigma |
| A83.01 | 5 µM | Tocris |
| Forskolin | 10 µM | Torcris |
| R-Spondin | 10% | Conditioned medium |
| WNT* | 30% WNT | Conditioned medium |
| Noggin* | 25 ng/mL | Conditioned medium |
| Y27632* | 10µM | Tocris |
| hES cell cloning recovery solution* | 1:1000 dilution | Stemgent |

** supplemented AdvDMEM/F12 (Gibco) contains 1M HEPES (Invitrogen), 1x L-Ultraglutamine (Invitrogen), 500mg/mL Primocin (invivogen) and 10000U/mL penicillin and streptomycine (Invitrogen).

**Medium formulation for non-canonical WNT stimulating conditions per mL.**

| **Component** | **Concentration** | **Brand** |
| --- | --- | --- |
| William’s-E |  | Gibco |
| EGF | 20 ng/mL | Peprotech |
| nicotinamide | 10nM | Sigma |
| sodium pyruvate | 6.3 mM | Invitrogen |
| sodium bicarbonate | 17 mM | Sigma |
| ascorbic acid | 0.2 mM | Sigma |
| glucose | 14 mM | Sigma |
| Ultraglutamine | 2mM | Invitrogen |
| dexamethasone | 0.1 µM | R&D systems |
| DKK1 | 100 ng/mL | R&D systems |
| ITS+ premix | 1x | BD Biosciences |
| R-Spondin | 10% | Conditioned medium |
| penicillin and streptomycine | 10000U/mL | Invitrogen |
| HEPES | 20mM | Invitrogen |

**Table S3. List of genes and primers used.**

**Gene** **Primer sequence (5’ à 3’)**

**Gene Primer sequence (5’à 3’)**

KRT7 F GGGGACGACCTCCGGAATAC
 R CTTGGCACGCTGGTTCTTGA
KRT19 F GCACTACAGCCACTACTACACGA
 R CTCATGCGCAGAGCCTGTT
HNF1B F TCACAGATACCAGCAGCATCAGT
 R GGGCATCACCAGGCTTGTA
HPRT1 F GCTATAAATTCTTTGCTGACCTGCG
 R CTTCGTGGGGTCCTTTTCACC
ALB F CTGCCTGCCTGTTGCCAAAGC
 R GGCAAGGTCCGCCCTGTCATC
GAPDH F CTTTTGCGTCGCCAGCCGAG
 R CCAGGCGCCCAATACGACCA
HNF4α F GTACTCCTGCAGATTTAGCC
 R CTGTCCTCATAGCTTGACCT
AQP1 F GGCCAGCGAGTTCAAGAAGAA
 R TCACACCATCAGCCAGGTCAT
SLC12A2 F ACCAAGGATGTGGTAGTAAGTGTGG
 R GGATTCTTTTTTCAACAGTGGTTGA
CD133 F CCTGGGGCTGCTGTTTATTA
 R ATCACCAACAGGGAGATTGC
SOX17 F ATACGCCAGTGACGACCAGA
 R TCCACGACTTGCCCAGCATC
FXR F GGGACAGAACCTGGAAGTGG
 R GCCTGTATACATACATTCAGCCA

A1AT F TGAGGAGAGCAGGAAAGGACA
 R CTCAGCCAGGGAGACAGG
GGT F TGGTGGACATCATAGGTGGGGA
 R ATGACGGCAGCACCTCACTT
SLC10A2 F GGTGGCCTTTGACATCCTCCC
 R GCATCATTCCGAGGGCAAGC
SOX9 F ACCAGTACCCGCACTTGCAC
 R GCGCCTTGAAGATGGCGTTG
EpCAM F GACTTTTGCCGCAGCTCAGGA
 R AGCAGTTTACGGCCAGCTTGT
CFTR F TGGCGGTCACTCGGCAATTT
 R TCCAGCAACCGCCAACAACT
LGR5 F GTCAGCTGCTCCCGAATCCC
 R TGAAACAGCTTGGGGGCACA
CYP3A4 F AGCAAAGAGCAACACAGAGCTGAA
 R CAGAGGTGTGGGCCCTGGAAT
TFF1 F ACAAGCTGCTGTACACGGACA
 R AAGTTTCCAGGGCCGGGCAAT
TFF2 F TCTGTCCTGCCTCCCTGATCCA
 R CTCTGGCACGTGAATCCCGGT
SOX4 F CCCAGCAAGAAGGCGAGTTA
 R CCTTCCAGTTCGTGTCCTCC

**Table S4. List of antibodies used.**

| **Antibody** | **Raised** | **Manufacturer-Reference** | **Dilution** |
| --- | --- | --- | --- |
| Albumin | Mouse - monoclonal | Sigma-Aldrich: A6684 | 1:500 |
| SOX9 | Mouse - monoclonal | ATLAS antibodies: 02712 | 1:200 |
| KRT-7 | Mouse - monoclonal | DAKO: M7018 | 1:100 |
| KRT-19 | Mouse - monoclonal | DAKO: M0888 | 1:100 |
| CFTR | Mouse - monoclonal | EMD Millipore Corp: MAB3484 | 1:100 |
| MUC-1 | Mouse | ThermoFisher Scientific:  MA5-14077 | 1:500 |
| SCTR | Rabbit | Abcam: AB234830 | 1:100 |
| Alexa Fluor 555 | Goat – polyclonal (anti-mouse) | ThermoFisher Scientific: A21422 | 1:200 |
| Alexa Fluor 488 | Goat – polyclonal (anti-rabbit) | ThermoFisher Scientific: A32731 | 1:200 |


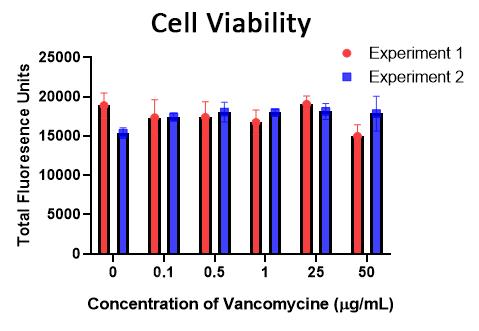


**Figure S1. Organoid Viability with vancomycin medium supplementation.**

AlamarBlue Fluorescence one week after initiation of bile organoid culture with different

concentrations of Vancomycin (n=2 experiments, per concentration 3 technical replicates). No difference in AlamarBlue activity was observed in these experiments, indicating similar cell viability and no harm of vancomycin in BCO cultures.


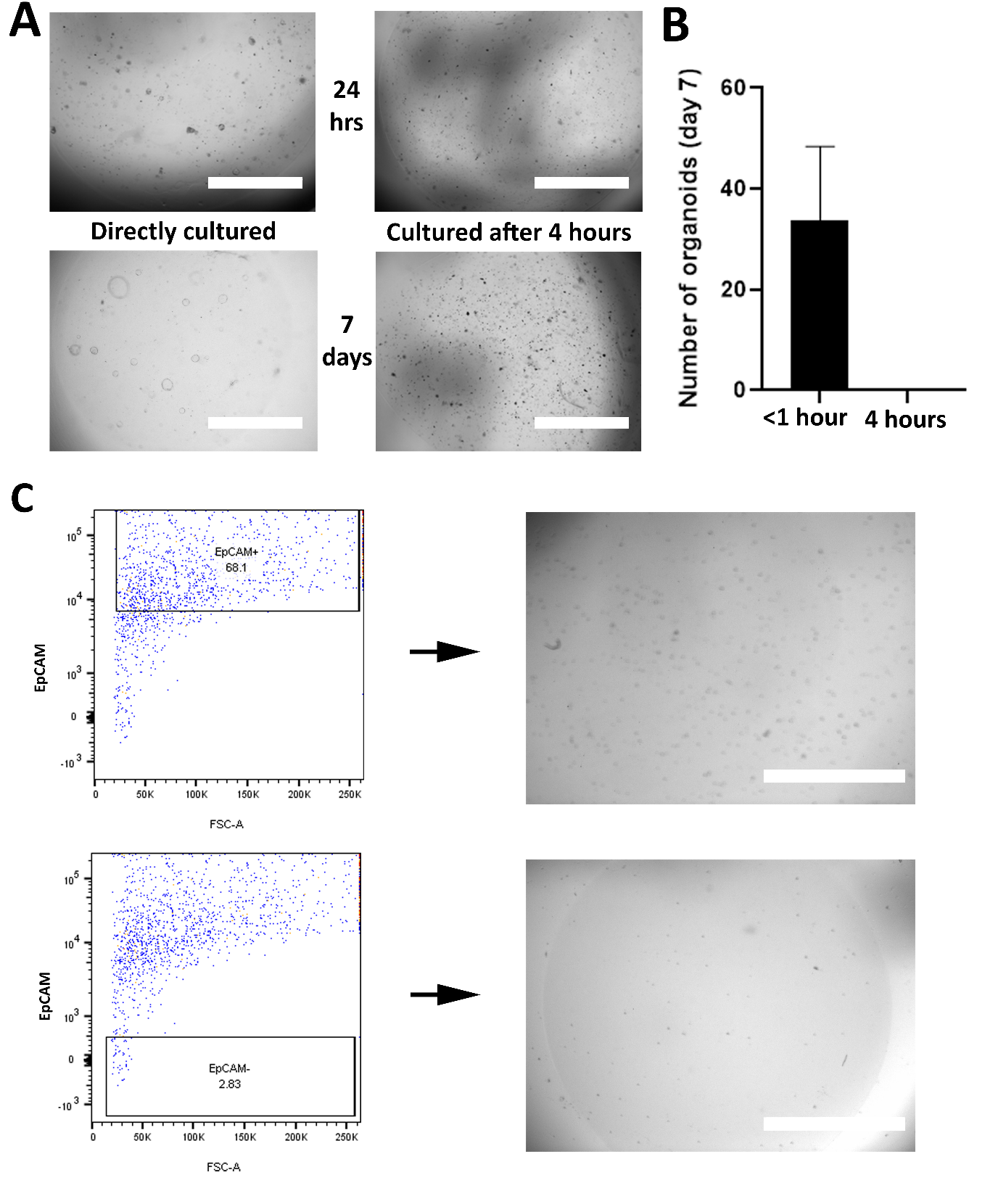


**Figure S2. Culture of ERCP-derived BCOs directly or after four hours of storage at 4°C.**

**(a)** On the left, bile samples were directly processed for organoid culture. While on the right, the bile samples were first stored at 4°C for four hours before being cultured as organoids. As shown in the bottom two pictures, bile samples directly cultured formed organoids after 7 days, while bile processed after 4 hours did not. Scale bars indicate 2mm. **(b)** Initiation of organoid cultures from ERCP-derived bile, cultured after < 1 hour or 4 hours of storage at 4°C, showing no outgrowth of organoids after a 4 hour storage period (n=3). * indicates a significant difference (p<0.05). **(c)** EpCAM^+^ and EpCAM^-^ flow cytometry sorted cells from bile resulted in no viable organoids after two weeks of culture.


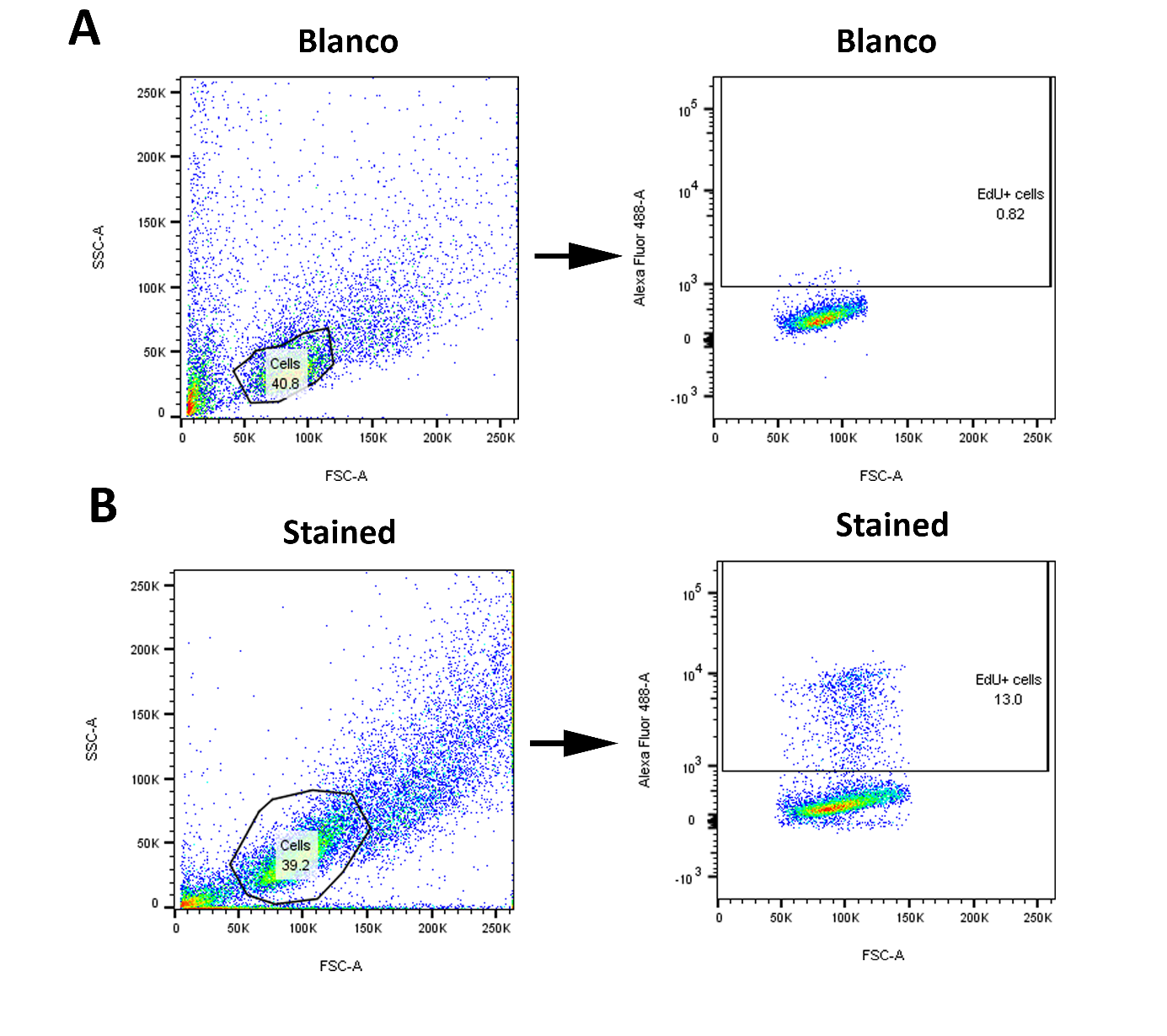


**Figure S3. Gating strategy of EdU staining in COs.**

**(a)** Top row unstained bile organoids (n=3, donors 1-3). First, cells were selected using forward-sideward scatter and next analysed on the Alexa-Fluor 488 spectrum. In **(b)** stained organoids are displayed.

**
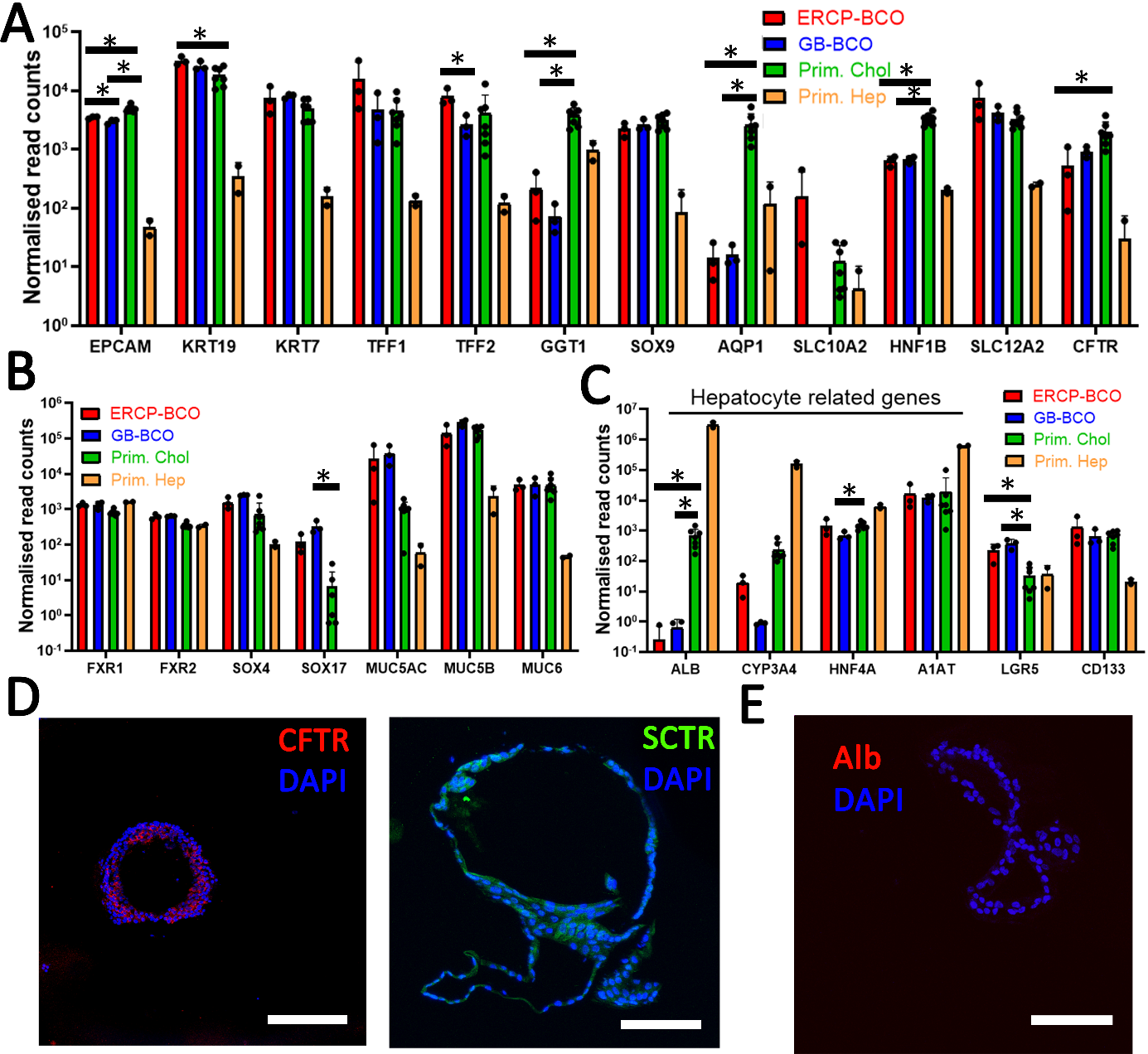

Figure S4. Characterization of BCOs.**

**(a-c)** Normalized read counts from RNAsequencing data for BCOs obtained from gallbladder-derived bile (n=3, BCO1-3) and ERCP-derived bile (n=3, BCO5-7) compared to expression-data of primary cholangiocytes obtained from common bile duct (n=7) as published^9^ and from 2D-cultured primary hepatocytes (n=2) as published by Schneeberger *et al.*^10^. All genes were significantly different expressed between primary cholangiocytes and hepatocytes (not indicated), except for TFF1, TFF2, SLC10A2, LGR5, FXR2, SOX4, SOX17, MUC5AC, and MUC6. All hepatocyte related genes in panel C were significantly lower in ERCP-BCOs and GB-BCOs compared to hepatocytes (not indicated). *indicates a significant difference between ERCP-BCOs, GB-BCOs and primary cholangiocytes (p<0.01). **(d)** Protein expression by immunofluorescence of the cholangiocyte markers: CFTR (red, left) and SCTR (green, right) with nuclei being counterstained (DAPI, blue) on BCOs (n=3, pictures displayed are from BCO5 and 7). **(e)** Protein expression by immunofluorescence shows absence of the hepatocyte marker: Albumin (red) in BCOs (n=3, BCO1, 5 and 7). Nuclei are counterstained with DAPI (blue). In both panels the scale bars indicate 200 µm.

**
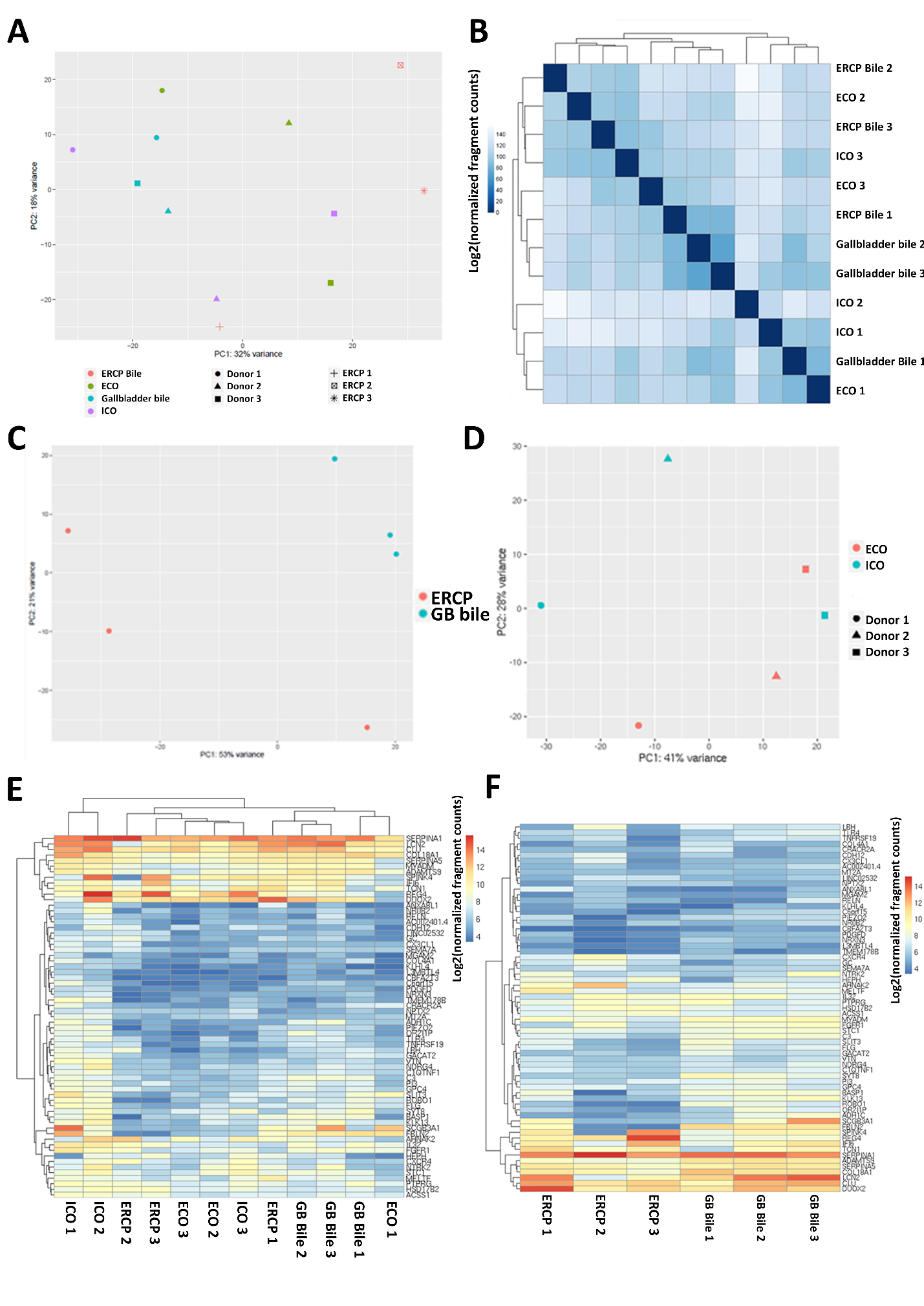
**

**Figure S5. Expression of DEgenes beween ICOs and ECOs on organoid samples.
(a)** Principal component analysis (PCA) of top 500 most variable genes between BCOs (ERCP-derived, n=3 BCO5-7 and gallbladder-derived, n=3, BCO1-3), displayed on all organoid sample types, showing no clear clustering pattern between sources, but overall a high correlation towards each other. **(b)** Heatmap**-**clustering showing sample-to-sample distances on the same samples as used in the PCA plot, showing no clear clustering on either source or donor. **(c)** Principal component analysis (PCA) of ERCP-derived BCOs and gallbladder-derived BCOs. **(d)** PCA plot of ICOs vs ECOs indicating that ICO3 overlaps with ECO gene-expression profiles. **(e)** Heatmap based upon ICO upregulated DEgenes on all 12 organoid samples, showing that ICO 1 and ICO 2 have an unique expression profile for ICO-specific genes compared to all other samples. **(f)** Heatmap of ICO-upregulated genes on all BCO samples, showing limited expression of these genes.

**
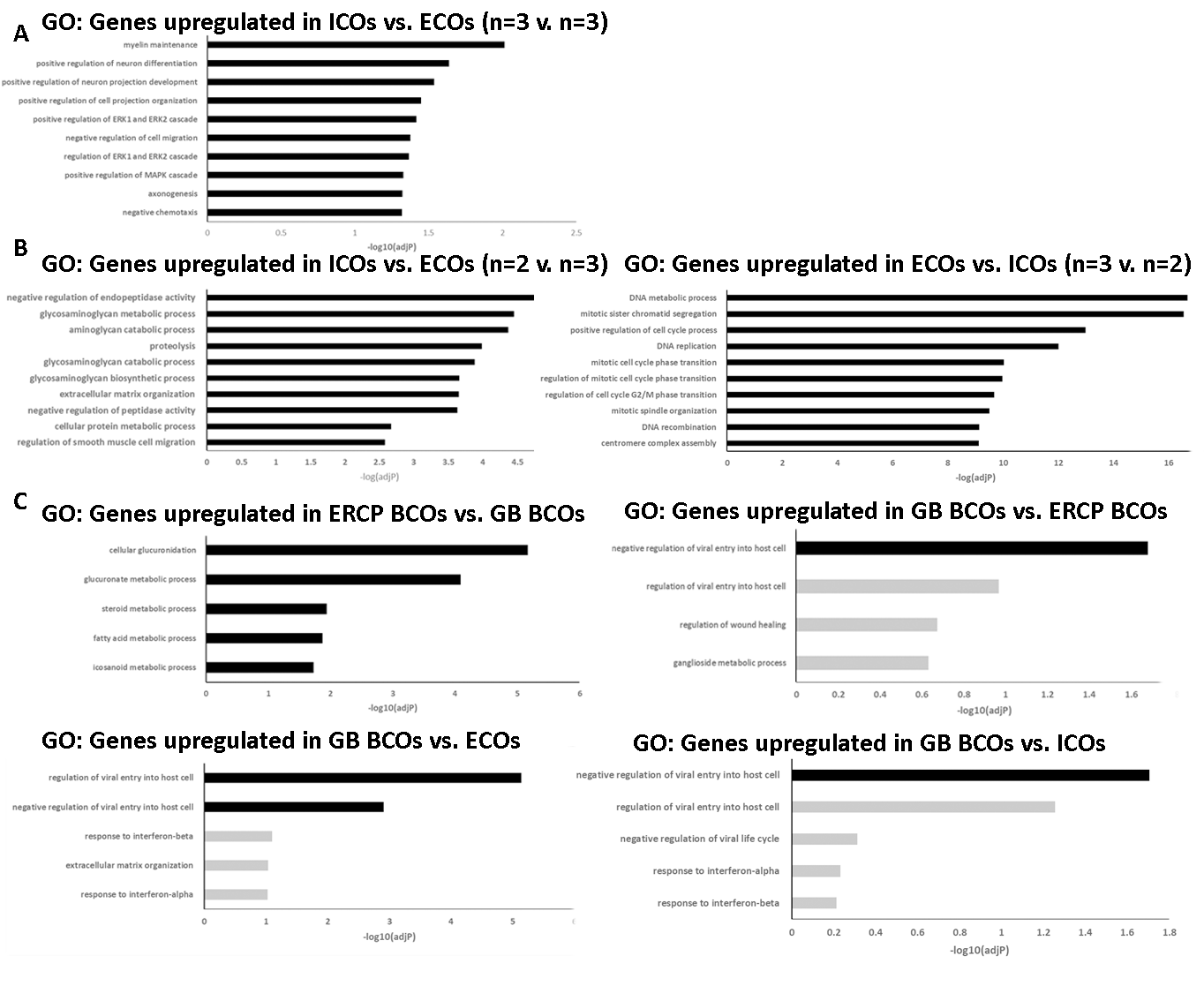
**

**Figure S6. Pathway analysis in cholangiocyte organoids from different sources.**

**(a)** Pathways analysis between ICOs and ECOs (n=3) revealed that certain pathways are enriched in ICOs compared to ECOs. No pathways were found to be significantly upregulated in ECOs compared to ICOs. **(b)** After exclusion of ICO 3, pathways analysis between ICOs (n=2) and ECOs (n=3) revealed that certain pathways are enriched in ICOs compared to ECOs. Interestingly, these analysis revealed that ECOs have significantly upregulated cell-proliferative pathways such as DNA replication and cell-cycle pathways. These results correspond to our EdU data (figure 3D) in which the two lowest proliferating ICO samples were ICO 1 and ICO 2 and ICOs were significantly less proliferative compared to ECOs. **(c)** Pathway analysis between different sources of BCOs showed a significant enrichment of metabolic-synthesis associated pathways in ERCP-derived BCOs compared to gallbladder-derived BCOs. No differences were found between ERCP BCOs and ICOs or ECOs. In contrast, GB-derived BCOs showed upregulation of viral-host interaction related pathways to both COs and ECOs as well as ERCP-derived BCOs. All experiments performed in Figure S6 are with donors 1-3.

**
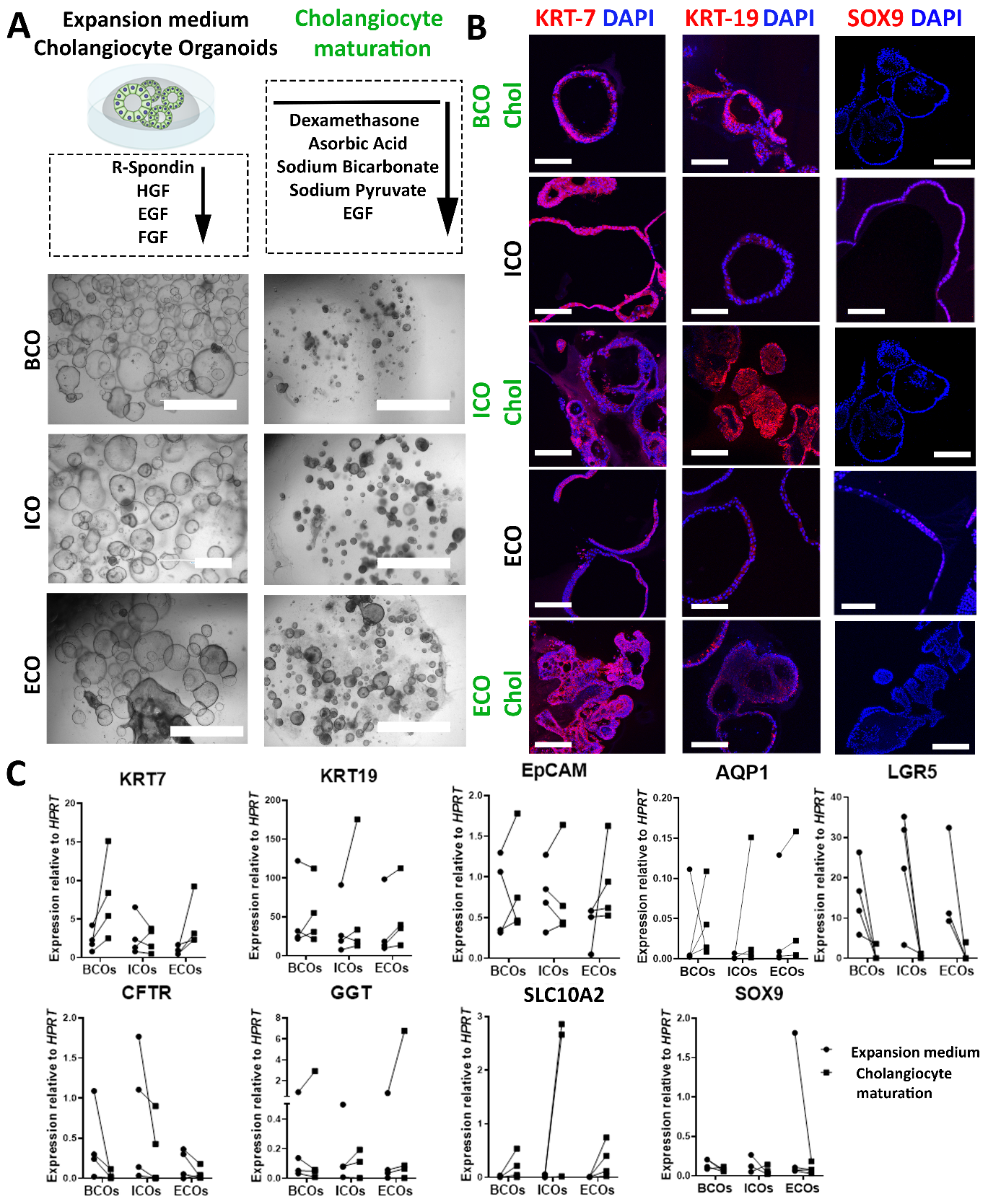

Figure S7. Cholangiocyte-organoids from all three sources can be partially matured using an established cholangiocyte-differentiation protocol.
(a)** Schematic overview and pictures of cholangiocyte-organoids (COs) from bile (BCO), intrahepatic biopsies (ICOs) or extrahepatic biopsies (ECOs) in either cholangiocyte maturation or in canonical-WNT stimulating conditions. Upon cholangiocyte maturation, 2D growth and smaller organoid size could be observed in multiple cultures in COs from all three sources. Scale bars indicate 2000µm. **(b)** Immunofluorescence images of protein expression in COs in differentiated and undifferentiated status for the cholangiocyte markers KRT-7, KRT-19 and SOX9. Images of BCOs in expansion medium for these proteins are displayed in Figure 2B. Upon cholangiocyte maturation, KRT-7 and KRT-19 expression seems more pronounced. While clear SOX9 expression could only be detected in one sample of an ICO upon cholangiocyte maturation. Scale bars indicate 100 µm. **(c)** Gene-expression by qRT-PCR relative to the housekeeper gene *HPRT1* for cholangiocyte maturation and paired organoids cultured in canonical-WNT stimulating conditions from all three sources (n=3), for either mature cholangiocyte markers (cytokeratin-*KRT-7*, *KRT19*, *CFTR*, *GGT*, *ASBT*, *EpCAM, AQP1* and *CFTR*) or stemness/cholangiocyte progenitor markers (*LGR5* and *SOX9*). ICOs, ECOs and BCOs had higher expression of *KRT7*, *ASBT*, *AQP1* and *KRT19* in a similar manner after maturation, while *EpCAM* and *GGT* gene-expression remained stable. Upon cholangiocyte maturation the expression of the WNT-target gene *LGR5* and the cholangiocyte progenitor marker *SOX9* was lower in all cultures from all sources. In contrast, the expression of mature cholangiocyte marker *CFTR* was lower in the cholangiocyte maturation protocol for organoids from all three sources. As forskolin (a cAMP-activator) was removed from the cholangiocyte maturation medium, the organoids cultured in these conditions are considerably smaller (Fig. S8A) compared to organoids in expansion medium. The downregulation of *CFTR* could be the result of less activation by forskolin and subsequent lack of swelling, as was previously published.^11^ However, overall our results indicate that no complete maturation is reached *in vitro* with this protocol, but in contrast to upregulation of hepatocyte-markers, here all organoid sources behave similar. All experiments performed in Figure S8 are with donors 1-3.


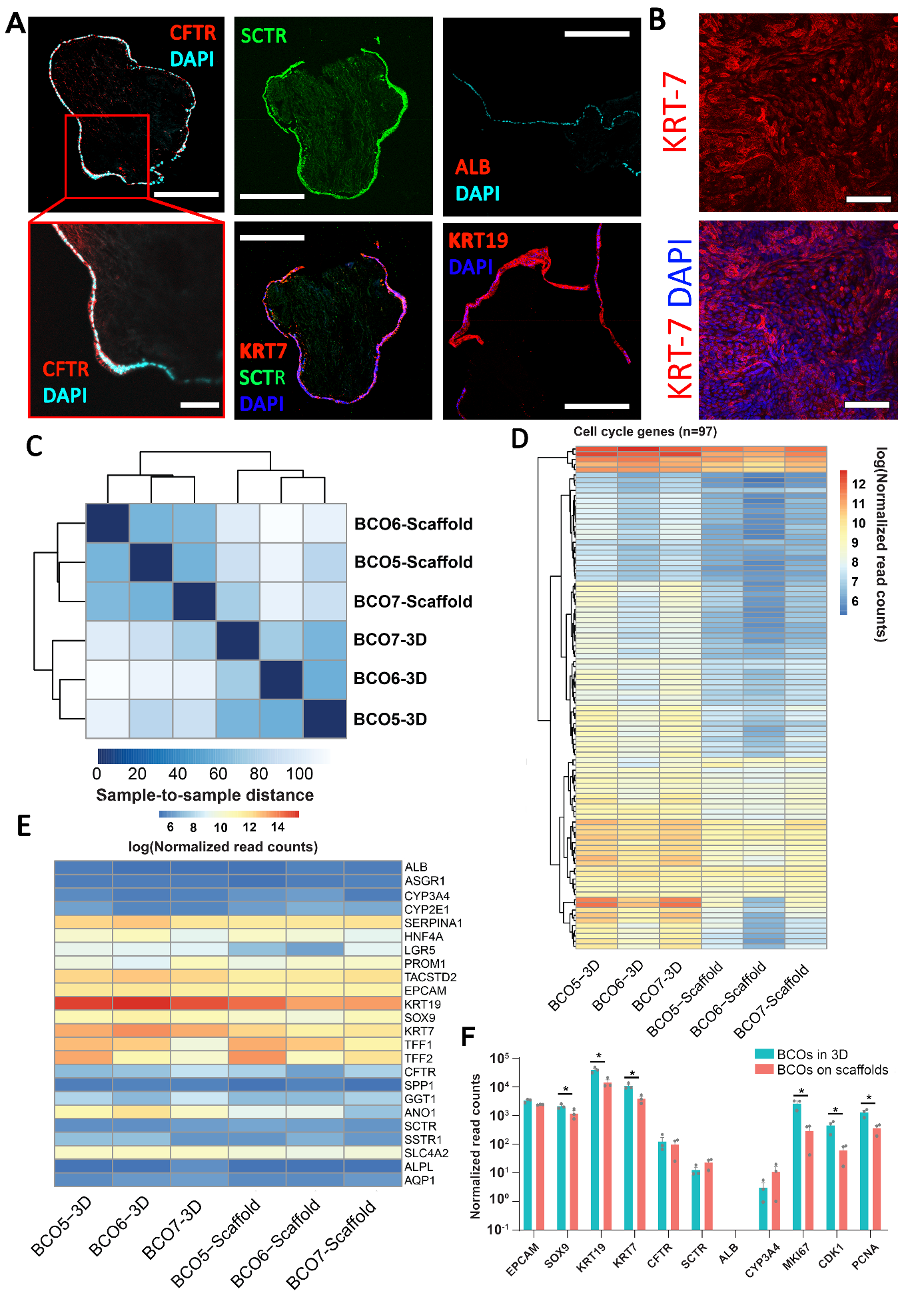

**Figure S8. BCOs can successfully recellularize EHBD-scaffolds as cholangiocyte-like cells.
(a)** Immunofluorescence staining of sections of BCO-recellularized scaffolds demonstrating presence of the mature cholangiocyte markers CFTR (red, upper/bottom left panel), SCTR (green, upper middle panel), KRT-7 (red, bottom middle panel), KRT-19 (red, bottom right panel) and absence of the hepatocyte related marker albumin (red, upper right panel). All sections are stained with DAPI (nuclei, cyan or dark blue). Scale bars indicate 200 µm. **(b)** Whole mount confocal images of immunofluorescence staining KRT-7 (red, upper panel) and KRT-7 and DAPI (nuclei, blue bottom panel) of repopulated EHBD scaffolds with BCOs, scale bars indicate 100µm. **(c)** Heatmap of sample-to-sample distances, showing a clear clustering based on the culture condition. **(d)** Heatmap showing the cell cycle gene expression from the samples in the 2 conditions. **(e)** Heatmap of the expression levels of a few selected hepatocyte and cholangiocyte markers between the 2 culture conditions. **(f)** Bar plot showing the expression of selected known cholangiocyte markers (*EPCAM*, *SOX9*, *KRT19*, *KRT7*, *CFTR*, and *SCTR*), hepatocyte markers (*ALB* and *CYP3A4*), and cell cycle related markers (*MKI67*, *PCK1*, and *PCNA*) in both conditions. *indicates a significant difference (p<0.05 adjusted for multiple testing).

**Supplementary references**

1. Broutier L, Andersson-Rolf A, Hindley CJ, *et al*. Culture and establishment of self-renewing human and mouse adult liver and pancreas 3D organoids and their genetic manipulation. Nat. Protoc. 2016;11:1724–1743.
2. Sampaziotis F, Justin AW, Tysoe OC, *et al*. Reconstruction of the mouse extrahepatic biliary tree using primary human extrahepatic cholangiocyte organoids. Nat. Med. 2017;23:954–963.
3. Huch M, Gehart H, Boxtel R van, *et al*. Long-term culture of genome-stable bipotent stem cells from adult human liver. Cell. 2015;1–14.
4. Soroka CJ, Assis DN, Alrabadi LS, *et al*. Bile-Derived Organoids From Patients With Primary Sclerosing Cholangitis Recapitulate Their Inflammatory Immune Profile. Hepatology. 2019 Sep;70(3):871-882.
5. Sampaziotis F, de Brito MC, Geti I, *et al*. Directed differentiation of human induced pluripotent stem cells into functional cholangiocyte-like cells. Nat Protoc. 2017 Apr;12(4):814-827
6. Sampaziotis F, de Brito MC, Madrigal P, *et al.* Cholangiocytes derived from human induced pluripotent stem cells for disease modeling and drug validation. Nat Biotechnol. 2015;33(8):845-852.
7. Verstegen MMA, Roos FJM, Burka K, *et al.* Human extrahepatic and intrahepatic cholangiocyte organoids show region-specific differentiation potential and model cystic fibrosis-related bile duct disease. Sci Rep. 2020 Dec 14;10(1):21900.
8. Willemse J, Roos FJM, Voogt IJ, *et al.* Scaffolds obtained from decellularized human extrahepatic bile ducts support organoids to establish functional biliary tissue in a dish. Biotechnol Bioeng. 2021 Feb;118(2):836-851.
9. Rimland CA, Tilson SG, Morell CM, *et al.* Regional differences in human biliary tissues and corresponding in vitro derived organoids. Regional Differences in Human Biliary Tissues and Corresponding In Vitro-Derived Organoids. Hepatology. 2021 Jan;73(1):247-267.
10. Schneeberger K, Sánchez-Romero N, Ye S, *et al.* Large-Scale Production of LGR5-Positive Bipotential Human Liver Stem Cells. Hepatology. 2020 Jul;72(1):257-270.

11. Van Mourik P, van Haaren P, Kruisselbrink E, *et al.* R117H-CFTR function and response to VX-770 correlate with mRNA and protein expression in intestinal organoids. J Cyst Fibros. 2020 Sep;19(5):728-732.
